# Generalizable Organic-to-Aqueous Phase Transfer of a Au_18_ Nanocluster with Luminescence Enhancement and Robust Photocatalysis in Water

**DOI:** 10.1021/acsnano.4c18197

**Published:** 2025-02-28

**Authors:** Zhongyu Liu, Yitong Wang, Weijie Ji, Xiaowei Ma, Christopher G. Gianopoulos, Sebastian Calderon, Timothy Ma, Lianshun Luo, Abhrojyoti Mazumder, Kristin Kirschbaum, Elizabeth C. Dickey, Linda A. Peteanu, Dominic Alfonso, Rongchao Jin

**Affiliations:** † Department of Chemistry, Carnegie Mellon University, Pittsburgh, Pennsylvania 15213, United States; ‡ Department of Chemistry and Biochemistry, University of Toledo, Toledo, Ohio 43606, United States; § Department of Materials Science and Engineering, Carnegie Mellon University, Pittsburgh, Pennsylvania 15213, United States; ∥ United States Department of Energy, National Energy Technology Laboratory, Pittsburgh, Pennsylvania 15236, United States

**Keywords:** atomically precise nanoclusters, phase transfer, water-soluble, photocatalysis, photoluminescence enhancement

## Abstract

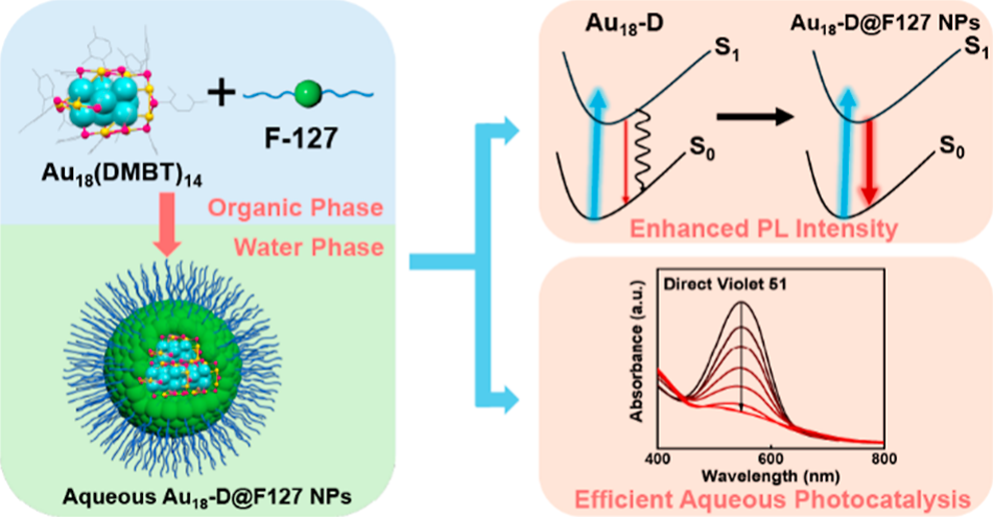

For the majority of gold nanoclusters (NCs), their water insolubility, low photoluminescence (PL) intensity, and less understood photostability are three critical factors that limit their application in the biomedical and photocatalysis fields. In this study, we report a polymer wrapping method for phase transfer of organic soluble NCs into aqueous phase without degrading the electronic and optical properties, and such materials are further demonstrated for robust photocatalysis in water. We first synthesized a Au_18_(DMBT)_14_ NC (DMBT = 2,4-dimethylbenzenethiolate) and found that the aromatic ligands confer a greatly enhanced antioxidation capability of the NC compared to the Au_18_(CHT)_14_ counterpart (CHT = cyclohexanethiolate), with the critical role of aromatic ligand interactions identified by X-ray crystallography. The organic soluble Au_18_(DMBT)_14_ was successfully transferred into the aqueous phase by an amphiphilic polymer (Pluronic F127, abbrev. F127) wrapping method, producing Au_18_-D@F127 nanoparticles [each containing a few NCs; Au_18_-D is an abbreviation for Au_18_(DMBT)_14_] with a 10-fold enhancement in PL intensity, and similar results were also obtained for Au_18_(CHT)_14_. This method is broadly applicable to various NCs, rendering their water solubility and significantly enhancing the PL intensity of otherwise weakly emissive gold NCs. The exceptional antioxidation stability of Au_18_(DMBT)_14_ enables the application of Au_18_-D@F127 NPs for the photocatalytic activation of persulfate ions and subsequent photodegradation of water pollutants efficiently.

## Introduction

Atomically precise metal nanoclusters (NCs) are an emerging type of materials with highly tunable optical properties.^1−13^ Due to their ultrasmall sizes (i.e., <3 nm), the strong quantum confinement effect leads to discrete energy levels in metal NCs,^1,14,15^ resulting in molecule-like absorption features and photoluminescence (PL). Through precise control, their absorption and PL profiles can be tailored to span the UV–vis to near-infrared II region.^16,17^ Due to their intriguing optical properties, metal NCs have been demonstrated as promising photosensitizers for photocatalysis, photo emitters for bioimaging fields, and in many other photorelated applications. Nevertheless, to achieve real-world applications, a few limitations still persist, as outlined below.

First of all, although many metal NCs have been reported, the majority of the known atomically precise metal NCs are only soluble in organic solvents (rather than in aqueous phase),^18−20^ which hinders their applications in biological and related systems, as well as electrochemical sensing of aqueous analytes. One way to tackle this problem is to synthesize aqueous NCs by using water-soluble ligands (e.g., γ-glutathione, cysteine, and mercaptobenzoic acid).^21−24^ Nevertheless, the resultant aqueous NCs are much harder to purify, analyze, and precisely engineer than organic soluble ones. Thus, it is highly desired to develop a universal method for transferring organic soluble NCs into the aqueous phase, which will be of paramount importance in order to further exploit the rich library of metal NCs for practical applications.

Second, high PL is important in many applications of metal NCs, but the PL intensity of many organic soluble gold NCs is quite low, with only a few exceptions.^25−30^ Most methodologies for PL enhancement, such as ligand engineering and heteroatom doping, that are used to enhance the PL intensity of gold NCs need to be studied on a case-by-case basis and require sophisticated synthetic processes.^31,32^ Thus, a general method to enhance their emission is urgently needed.

Third, the photostability of many NCs is not well studied yet, but it is of critical importance in photocatalysis.^33−41^ A recent study found that NCs protected by aromatic ligands are much more photostable than those protected by alkyl thiolates because aromatic ligands are much less likely to form radicals under light irradiation.^42^ Hence, aromatic ligands might be a better option for synthesizing metal NCs for optics and related applications.

To address the above issues, herein, we report a general strategy for transferring organic soluble NCs into aqueous solutions for achieving an enhancement of PL and robust photocatalytic activity in water. We demonstrate the methodology with the Au_18_(DMBT)_14_. The aromatic ligand (DMBT) introduces strong interligand π–π interactions and C–H−π interactions on Au_18_(DMBT)_14_, leading to a significant enhancement of its antioxidation capability and photostability. Using a Pluronic F127 assisted method, the organic-soluble Au_18_(DMBT)_14_ is successfully transferred into the aqueous phase without changing its original structure and electronic properties. The obtained aqueous Au_18_-D@F127 nanoparticles (∼2 nm) exhibit a 10-fold enhancement in PL intensity compared to the original organic soluble Au_18_(DMBT)_14_. Moreover, this method is universal for transferring thiolate-protected gold NCs and is able to turn barely emissive NCs into moderately emissive ones. The synthesized Au_18_-D@F127 nanoparticles are further utilized to efficiently activate persulfate ions for the aqueous photocatalytic degradation of organic pollutants.

## Results and Discussion

### Synthesis and Characterization of Au_18_(DMBT)_14_

**Figure 1:**
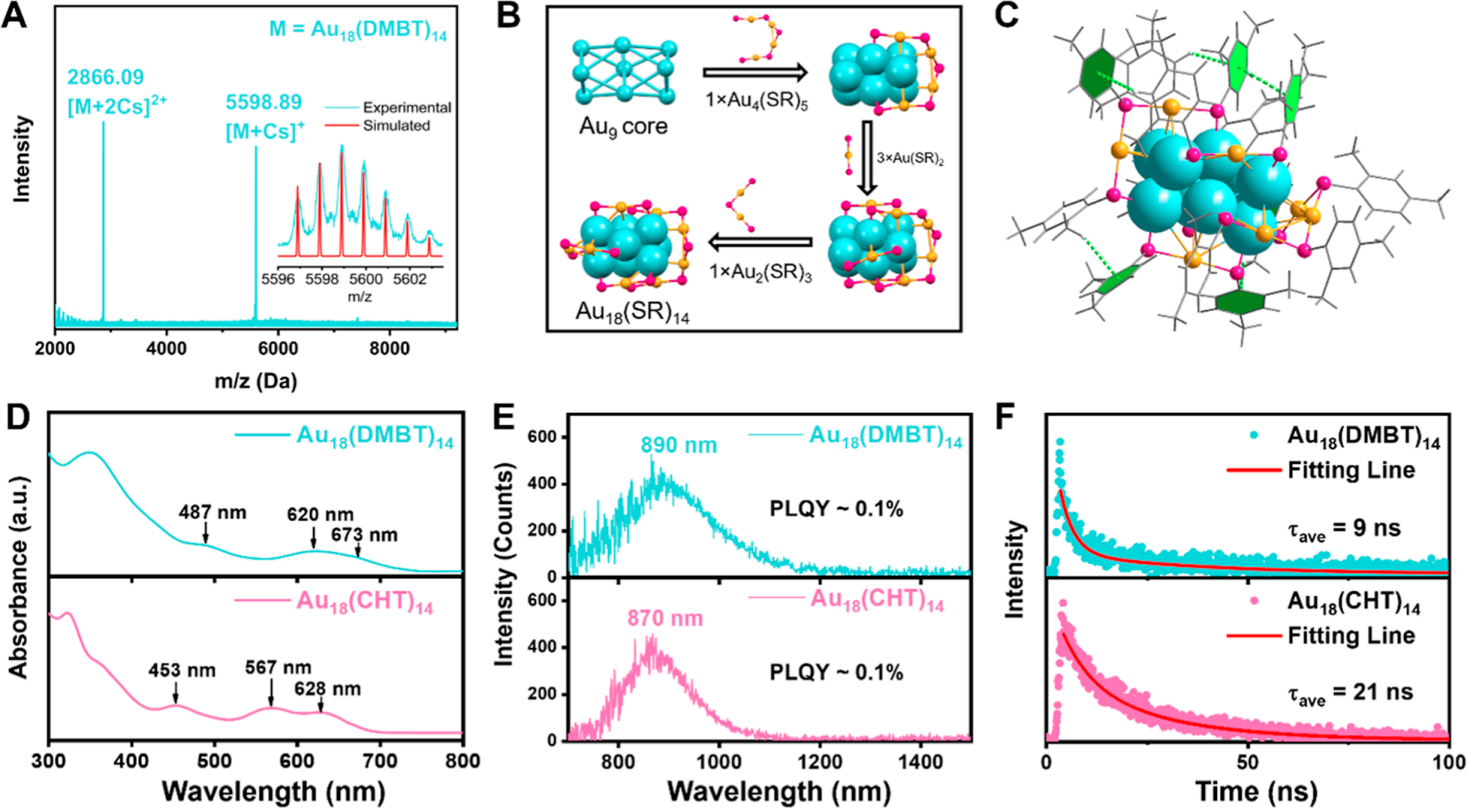
(A) ESI mass spectrum of Au_18_(DMBT)_14_ (CsOAc was added to facilitate ESI analysis); inset shows the experimental isotope pattern (blue profile) of *m*/*z* at 5598.89 and the theoretical pattern (red). (B) Anatomy of the X-ray structure of Au_18_(DMBT)_14_ NC. (C) Illustration of intramolecular π–π interaction and C–H−π interaction in Au_18_(DMBT)_14_. (D) UV–vis absorption spectra of Au_18_(DMBT)_14_ (upper panel) and Au_18_(CHT)_14_ (bottom panel). (E) PL spectra of Au_18_(DMBT)_14_ (upper panel) and Au_18_(CHT)_14_ (bottom). (F) PL decay curves of Au_18_(DMBT)_14_ (upper panel) and Au_18_(CHT)_14_ (bottom).

The synthesis of Au_18_(DMBT)_14_ involved a typical method of Au(I) reduction (see details in the Supporting Information). Briefly, Au(I)-DMBT polymers were first prepared and subsequently reduced under basic and ice-cold conditions to attenuate the reducing power of NaBH_4_. After 8 h, the target Au_18_(DMBT)_14_ NC was isolated from the crude product using thin-layer chromatography (TLC). The pure Au_18_(DMBT)_14_ appears as a green band on the TLC plate (Figure S1). The molecular formula was determined by electrospray ionization mass spectrometry, with cesium acetate added to form charged adducts. As shown in Figure 1A, two peaks at *m*/*z* 2866.09 and 5598.89 are observed, which are identified as [Au_18_(DMBT)_14_+2Cs]^2+^ (theoretical *m*/*z* = 2865.90) and [Au_18_(DMBT)_14_+Cs]^+^ (theoretical *m*/*z* = 5598.90), respectively.

Dark green crystals (Figure S2) of Au_18_(DMBT)_14_ were obtained by diffusion of ethanol into a dichloromethane solution of Au_18_(DMBT)_14_. The structure of Au_18_(DMBT)_14_ was solved by single-crystal X-ray crystallography. Detailed crystallographic information is given in Tables S1 and S2. The overall structure of Au_18_(DMBT)_14_ was found to be similar to that of the previously reported Au_18_(CHT)_14_.^43,44^ In both cases, a face-fused bioctahedral Au_9_ kernel (or three Au_3_ layers in hexagonal close-packing) is seen, and the surface-protecting shell includes three monomeric Au(DMBT)_2_, one dimeric Au_2_(DMBT)_3,_ and one tetrameric Au_4_(DMBT)_5_ staple motifs (Figure 1B). Nevertheless, the arrangement of carbon tails on the surface of Au_18_(DMBT)_14_ is completely different from that of Au_18_(CHT)_14_. The aromatic nature of the DMBT ligand exhibits interligand π–π and C–H−π interactions (Figure 1C), which help to enhance the shielding effect and block external attacks. This contributes to the enhanced stability of the NCs.^45^

Although structurally similar, Au_18_(DMBT)_14_ and Au_18_(CHT)_14_ exhibit some differences in their absorption spectra (Figure 1D). The spectrum of Au_18_(DMBT)_14_ shows a major peak at 620 nm and an inconspicuous shoulder at 673 nm on the long wavelength end, whereas Au_18_(CHT)_14_ displays two distinct absorption peaks at 567 and 628 nm. The ∼50 nm redshift can be attributed to the aromatic nature of the protecting DMBT ligand, which enhances electron delocalization. Typically, aromatic ligands can cause a redshift of <20 nm in electronic transitions;^46,47^ herein, a shift as large as 50 nm is quite rare. The optical gap of Au_18_(DMBT)_14_ is determined to be 1.63 eV (Figure S3A) by extrapolating the lowest energy absorption peak to zero absorbance, which is 0.14 eV smaller than that of Au_18_(CHT)_14_ (Figure S3B).

The PL emission spectrum (Figure 1E, upper panel) of Au_18_(DMBT)_14_ shows a single emission peak at 890 nm, with the quantum yield determined to be 0.1% by a relative method using Au_25_(PET)_18_^–^ (PLQY = 1.0%, PET = 2-phenylethanethiolate), as shown in ref (46). The PL emission spectrum of Au_18_(CHT)_14_ (Figure 1E, bottom panel) presents a similar PLQY (0.1%) and a similar profile but with a 20 nm blueshift compared to Au_18_(DMBT)_14_. The average PL lifetimes of Au_18_(DMBT)_14_ and Au_18_(CHT)_14_ (Figure 1F) are determined to be 9 and 21 ns, respectively. Detailed fitting results of the lifetimes are shown in Table S3. Overall, the low PL quantum yields observed in both Au_18_(DMBT)_14_ and Au_18_(CHT)_14_ indicate that nonradiative relaxation channels dominate the energy dissipation in both cases. These channels are barely affected by the ligand’s carbon tail modification, suggesting that the low PLQY is related to the staples and the core.

### Water-Soluble Au_18_-D@F127 Nanoparticles and Photoluminescence Enhancement

**Figure 2:**
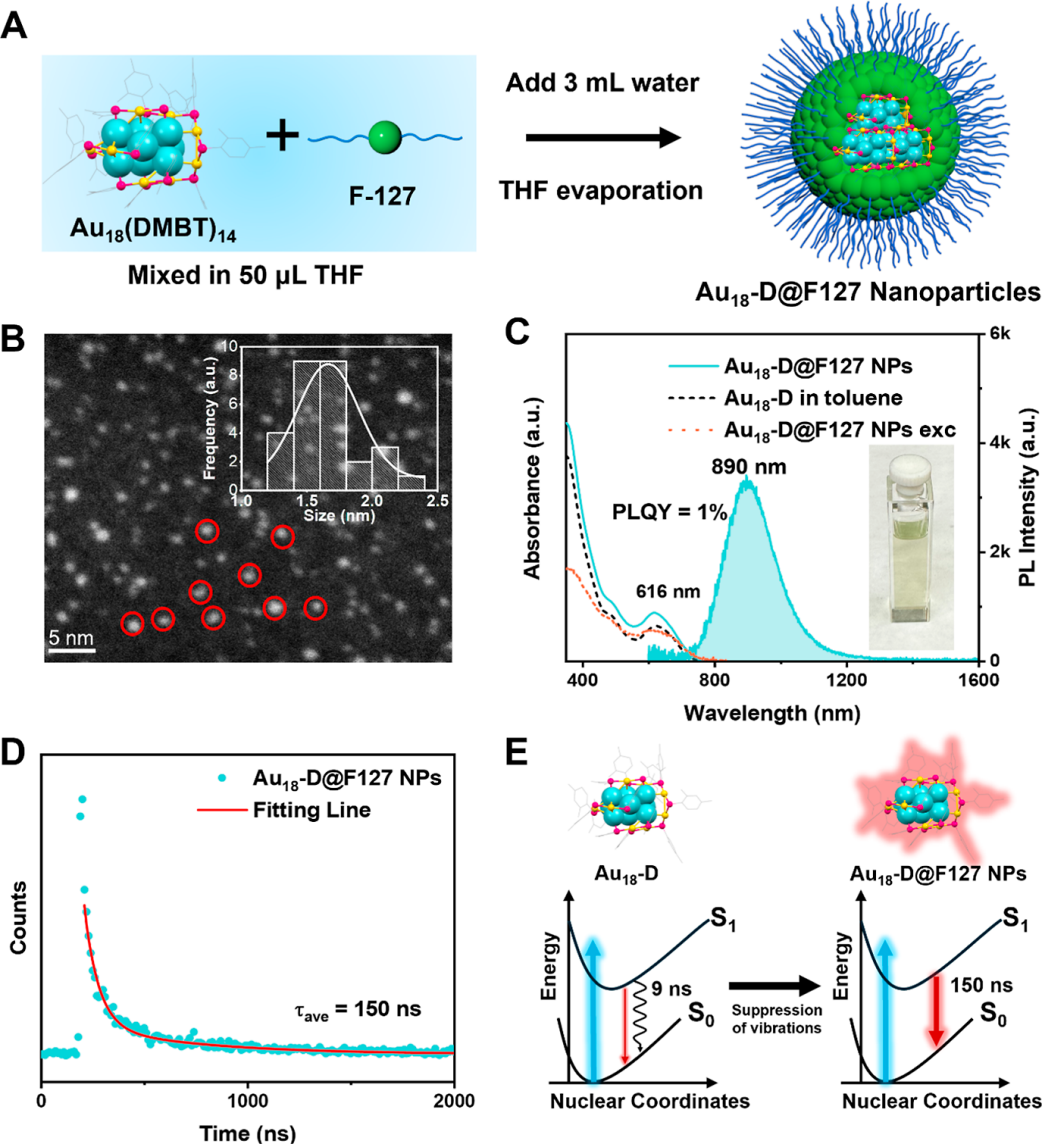
(A) Schematic formation process of Au_18_-D@F127 NPs. (B) Scanning transmission electron microscopy (STEM) image of Au_18_-D@F127 NPs and their size distribution (shown in the inset). (C) Absorption (solid line) and PL (shaded area) spectra of Au_18_-D@F127 NPs in deaerated D_2_O (with N_2_). The dashed black line is the absorption spectrum of Au_18_-D in toluene. The dotted red line is the excitation spectrum for PL at 890 nm. (For PL measurements: excitation at 400 nm with 0.2 optical density (OD), slit width 8 nm, and emission slit 8 nm.) (D) PL decay curve of Au_18_-D@F127 NPs in D_2_O (the red line is the fitting result). (E) Schematic illustration of excited-state dynamics of Au_18_-D and Au_18_-D@F127 NPs.

Many applications require water-soluble NCs, such as bioimaging and chemical sensing. Here, we propose a facile and universal method (Figure 2A) to efficiently transfer organic soluble gold NCs into a water phase without altering their original optical properties. To achieve that, we introduce an amphiphilic polymer, Pluronic F127 (F127), which contains hydrophilic blocks of ethylene oxide on the two ends of the polymer and a hydrophobic block in the middle. F-127 first mixed in 50 μL of tetrahydrofuran (THF) containing Au_18_(DMBT)_14_ [Au_18_-D hereafter, where “D” is to differentiate from the Au_18_(CHT)_14_ counterpart]. The mixture was then added to 3 mL of water, and the residual THF was removed by blowing nitrogen into the mixture for 1 h at 35 °C. Finally, water was added again to bring the volume of **Au**_**18**_**-D@F127** solution back to 3 mL.

Figure 2B shows a representative STEM image of the Au_18_-D@F127 NPs (<2.5 nm core diameter). Interestingly, after the introduction of F127, we did not observe large aggregation of gold NCs; instead, only sparse and small NPs were seen, and the gold core was confirmed by elemental analysis (Figure S4). This suggests that each Au_18_-D@F127 nanoparticle contains only up to a few discrete Au_18_(DMBT)_14_ NCs (1 nm), which differs from the previously reported assembly of thousands of NCs into supraparticles of tens of nm in size.^48,49^

The UV–vis absorption spectrum (Figure 2C, blue line) of Au_18_-D@F127 NPs in D_2_O is consistent with the absorption spectrum of Au_18_-D in organic solvent, indicating that polymer wrapping of NCs and self-aggregation do not alter the original structure and electronic properties of Au_18_-D. No strong scattering was observed here, confirming that the size of the NP is small. Interestingly, the emission intensity (Figure 2C, shaded area) of Au_18_-D@F127 was found to be 10 times higher than that of the original Au_18_-D NC. The PL excitation spectrum (Figure 2C, red line) of Au_18_-D@F127 matches the absorption profile, confirming that the observed emission originates from the HOMO–LUMO transition in Au_18_-D. Time-resolved PL measurements (Figure 2D) reveal that the average PL lifetime of Au_18_-D@F127 is 15 times longer than that of Au_18_-D (the organic one). The observed longer lifetime indicates that the overall electron relaxation rate of Au_18_-D is slower in Au_18_-D@F127. Since the PLQY and lifetime scale up by a similar factor and the dominant energy relaxation pathway here is the nonradiative channel, the enhancement of PL can be primarily ascribed to the suppression of vibrations. This suppression may stem from the stacking of adjacent NCs and interactions between F127 and Au_18_-D.^50^ We tried the same protocol for Au_18_(CHT)_14_, and the transfer was successful, too, and a similar enhancement of PL was observed (Figure S5 and Table S4). The similar enhancement of the two Au_18_ NCs further proves that the core of Au_18_ is the critical factor that limits the PL quantum yield.

**Figure 3:**
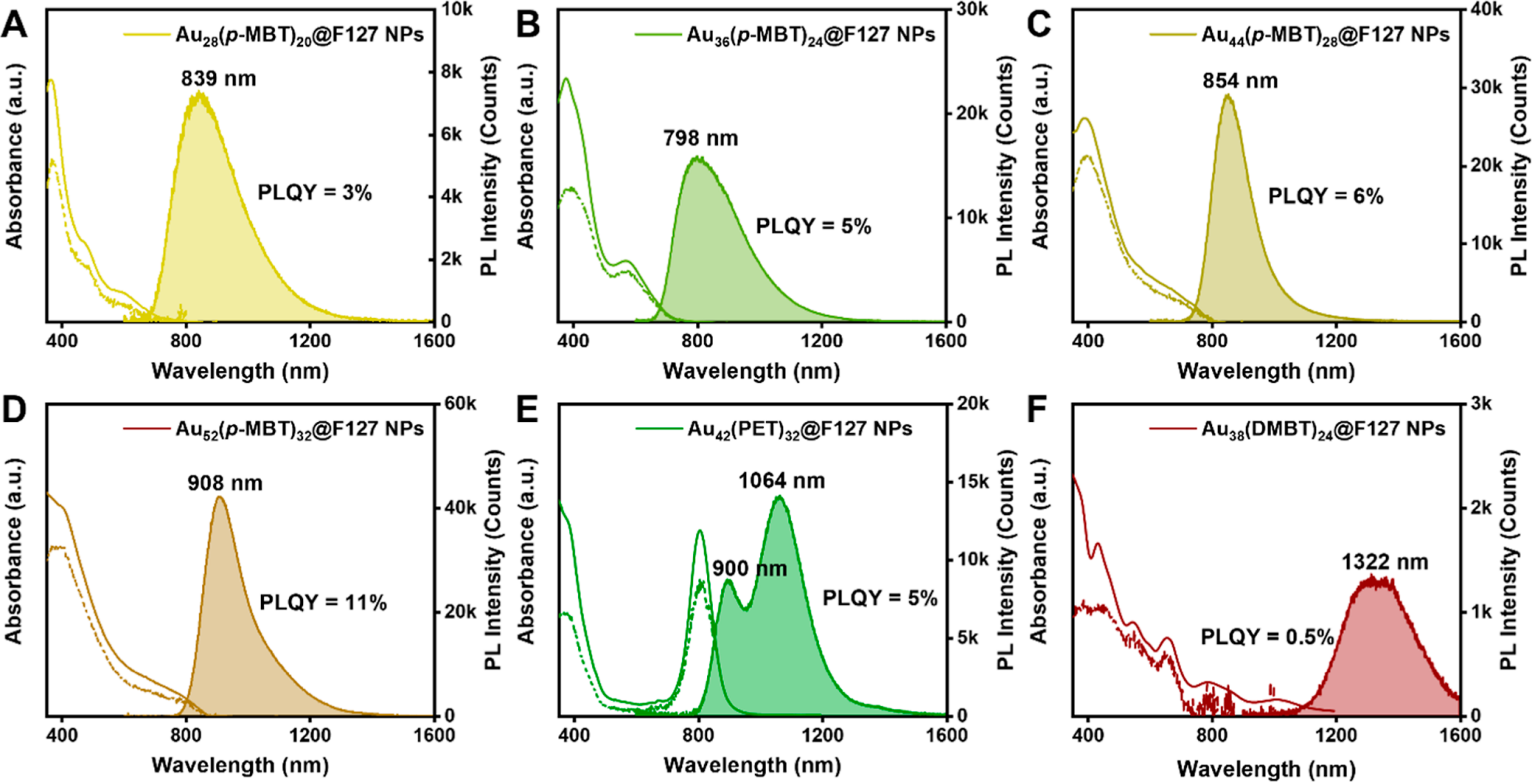
Absorption (solid lines) and PL emission (shaded areas) spectra of F127 wrapped NCs. (A) Au_28_(*p*-MBT)_20_, (B) Au_36_(*p*-MBT)_24_, (C) Au_44_(*p*-MBT)_28_, (D) Au_52_(*p*-MBT)_32_, (E) Au_42_(PET)_32,_ and (F) Au_38_(DMBT)_24_ in deaerated D_2_O (with N_2_). Dashed lines represent the PL excitation spectra. (For PL measurements: excitation at 400 nm with 0.2 OD, slit width 8 nm, and emission slit 8 nm).

**Figure 4:**
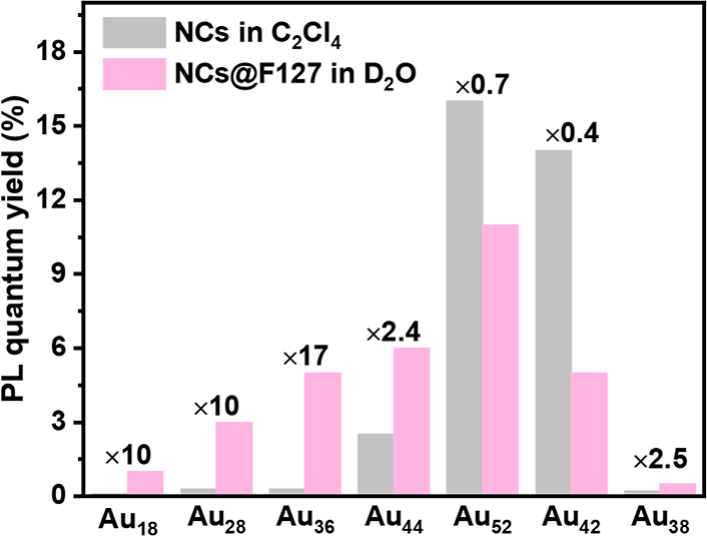
Comparison of PLQY for six NCs in C_2_Cl_4_ (gray bars) and after transfer into D_2_O (pink bars). For Au_18_, its PLQY in organic solution is very low (∼0.1%) and thus barely discernible in the bar graph.

To test the universality of the F127 wrapping strategy, we applied this method to various gold NCs, including Au_28_(*p*-MBT)_20_, Au_36_(*p*-MBT)_24_, Au_44_(*p*-MBT)_28_, Au_52_(*p*-MBT)_32_, Au_42_(PET)_32,_ and Au_38_(DMBT)_24_ (where, *p*-MBT = 4-methylbenzenethiolate). As shown in Figure 3A–F, all six NCs were successfully transferred into aqueous phase without changing their original absorption profiles.^28,32,47,51^ The PL excitation spectra (Figure 3A–F) of all six NCs match perfectly with their aqueous absorption spectra, indicating that all emissions originate from the first excited states of these NCs. The PL spectra of the six NCs in the aqueous phase also retained the same profiles as those of their organic counterparts (Figure S6). Notably, the ratio of the two emission peaks in Au_42_ changed slightly after transferring from the organic phase to water (Figures 3E and S6E). A similar phenomenon was observed when embedding Au_42_ into polystyrene films, in which the 900 nm emission peak of Au_42_ completely disappeared due to extensive and strong interparticle interactions in the close packed state.^28^ Therefore, the slight decrease in the 900 nm emission peak indicates that the F127 nanoparticles trap only up to a few individual NCs, because extensive assembly would lead to a complete disappearance of the 900 nm PL peak. The indication of one to several NCs in the polymer micelle is consistent with the STEM imaging results. Interestingly, in terms of PL intensity, a strong PL enhancement was observed for Au_28_(*p*-MBT)_20_, Au_36_(*p*-MBT)_24_, Au_44_(*p*-MBT)_28_, and Au_38_(DMBT)_24_ (Figure 4). For example, the PL intensity of Au_36_(*p*-MBT)_24_ increased nearly 20-fold after the aqueous nanoparticles were formed; thus, our strategy shows a great potential for exploiting the potential of the aggregation-induced emission (AIE) type of gold NCs.^23,52^ In contrast, the slight decrease in PL intensity for Au_52_(*p*-MBT)_32_ and Au_42_(PET)_32_ may be attributed to their one-dimensional rod-like structure, which exhibits stronger exciton–exciton annihilation, possibly leading to PL quenching. This quenching effect is also observed in the high-concentration organic solution of these two NCs.

Overall, this F127 wrapping methodology is significant, in that it can turn barely emissive NCs into moderately emissive NCs without changing their original optical and electronic properties, providing a promising method for exploring the aqueous phase applications of currently known gold NCs. This is in contrast with the organic-to-aqueous phase transfer of quantum dots or organic dyes, which inevitably results in strong quenching of the PL emission intensity. Therefore, gold NCs show better performance in retaining PL emission during oil-to-water phase transfer, paving the way for future applications that require aqueous NCs. We also tested the stability of Au_18_-D@F127 NPs in a simulated biofluid and found a good stability (Figure S7). A poor solvent-induced AIE test of Au_18_-D was performed by using a methanol/THF mixture (Figure S8). The results show a 3-fold increase in emission at a methanol-to-THF ratio of 10:1; thus, the solvent method is less effective than the F127 wrapping strategy (10X enhancement). The wide emission range (700 to 1600 nm) and enhanced PL intensity of gold NCs may open possibilities for the use of these materials in advanced diagnostic imaging, targeted therapy, and real-time monitoring of biological processes.^53−55^ Below we demonstrate an application in aqueous photocatalysis.

### Photocatalytic Degradation of Organic Pollutants Using Au_18_-D@F127 Nanoparticles

Atomically precise NCs have been reported in various photocatalytic processes, such as water splitting, organic pollutant degradation, CO_2_ reduction, etc.^34,56^ However, a major concern is the insufficient photostability of NCs due primarily to the oxidation of NCs by photoinduced holes.^57^ Photocatalytic oxidation of organic ligands that protect Au NCs was also reported to trigger the aggregation of Au NCs and their gradual transformation into plasmonic NPs.^58^ Hence, NCs with strong antioxidation capability are critically needed for stable photocatalytic reactions.

**Figure 5:**
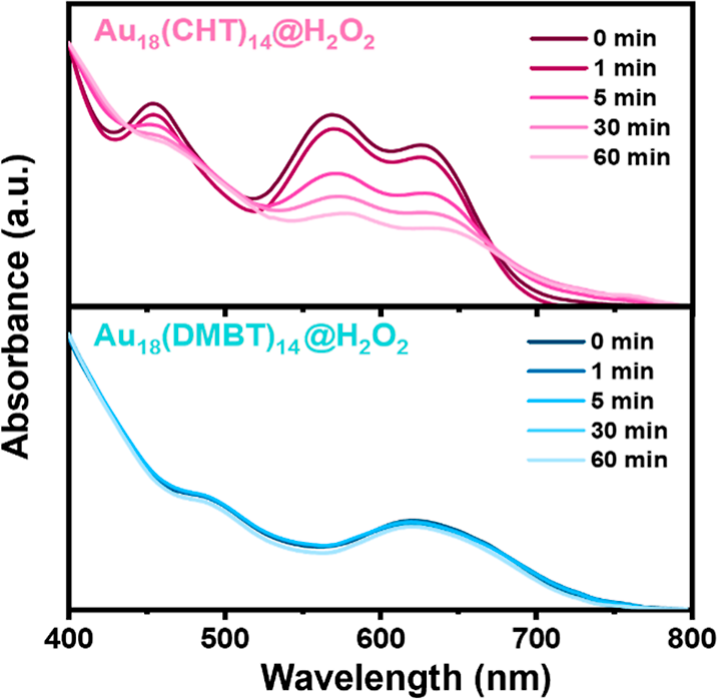
Antioxidation stability of Au_18_(CHT)_14_ (upper panel) and Au_18_(DMBT)_14_ (bottom panel) during the treatment with H_2_O_2_ (monitored by time-dependent optical absorption).

We first tested the oxidation stability of Au_18_ NCs. In the test, Au_18_ NCs were dissolved in 1 mL of THF and mixed with 2 mL of 30% H_2_O_2_ aqueous solution. As shown in Figure 5, Au_18_(CHT)_14_ decomposed rapidly after the addition of H_2_O_2_, whereas Au_18_(DMBT)_14_ remains intact during the test, indicating that Au_18_(DMBT)_14_ possesses an excellent antioxidation stability. The enhanced antioxidation stability of Au_18_(DMBT)_14_ may originate from the π–π interactions and C–H−π interactions of its aromatic ligand shell.^59,60^ A similar trend was also previously observed in Au_25_ NCs.^45,61^ In addition, we rationalize that in an aromatic ligand, the lone pair of electrons on the S atom can delocalize into the π-system of the benzene ring. The reduced electron density on the S atom disfavors radical attack to the S atom and the Au–S linkage; thus, Au_18_(DMBT)_14_ becomes more resistant to radical attacks compared to the case of nonaromatic thiolate protection.

**Scheme 1:**
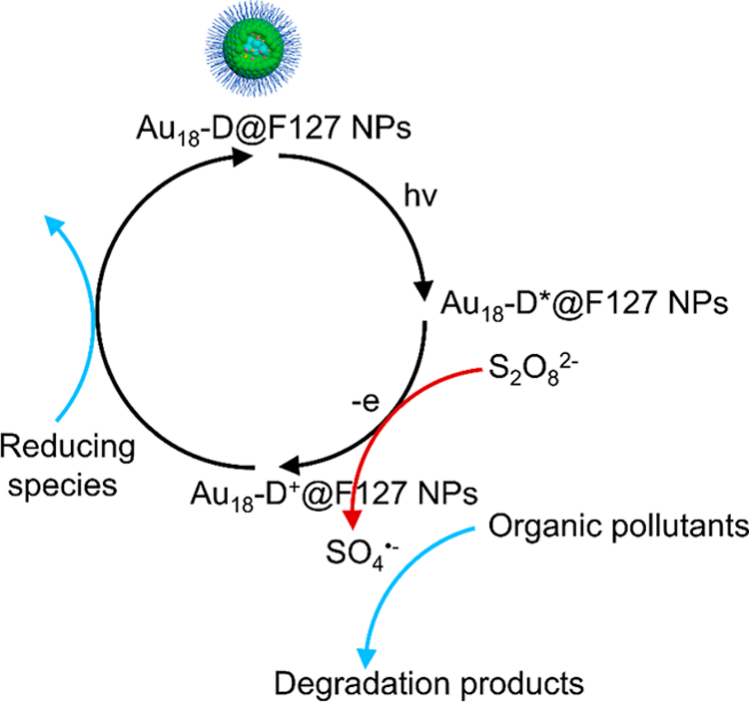
Photocatalytic Degradation of Organic Pollutants Using Au_18_-D@F127 Nanoparticles as a Catalyst

The remarkable antioxidation capability of Au_18_(DMBT)_14_ and prolonged excited-state lifetime of Au_18_-D after F127 coating render such NCs promising for photocatalysis. Combined with the excellent aqueous solubility of Au_18_-D@F127 NPs, all of these properties inspired us to apply Au_18_-D@F127 NPs for the photocatalytic degradation of organic pollutants. Ammonium persulfate was chosen as the oxidative agent because it can be easily activated to form sulfate radicals, which have a high oxidation potential (*E*_o_ = 2.60 V) and long radical lifetime (∼40 μs).^62^ We propose a photocatalytic cycle, illustrated in Scheme 1. Upon irradiation, electrons in the ground state of Au_18_-D@F127 are excited to the photoexcited state (Au_18_-D*@F127). The excited Au_18_-D*@F127 then interacts with an adsorbed persulfate ion, transferring an electron to the persulfate ion and forming an oxidized Au_18_-D^+^@F127 at the ground state and a sulfate radical. This sulfate radical diffuses out and reacts with organic pollutants. Finally, the oxidized Au_18_-D^+^@F127 reacts with a reducing species derived from organic pollutants to regenerate the original catalyst. We have measured the differential pulse voltammetry of Au_18_-D in the organic phase and Au_18_@F127 in the water phase (Figure S9). The water phase experiment is less informative due to poor conductivity of the F127 shell, but the organic phase result shows the first oxidation wave at +0.83 V (vs AgQRE) and the first reduction wave at −0.86 V, thus the oxidization by S_2_O_8_^2–^ can occur. The electrochemical gap of Au_18_-D is also consistent with the optical gap (1.63 eV).

**Figure 6:**
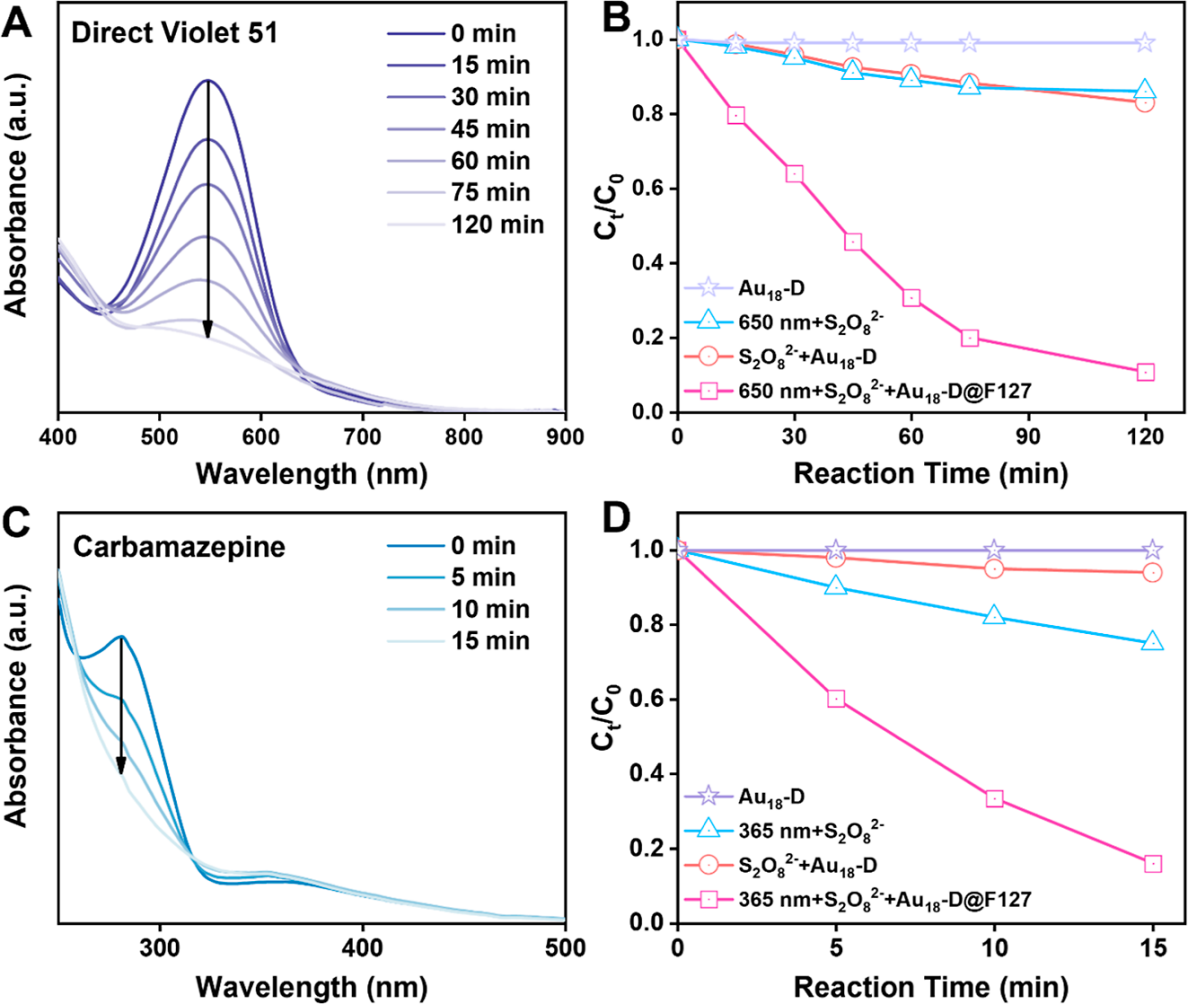
(A) UV–vis spectra of DV51 during photocatalytic degradation. (B) Comparison of photocatalytic degradation efficiency of DV51 with various control groups (data adapted from panel A). (C) UV–vis spectra of CBZ during photocatalytic degradation. (D) Comparison of photocatalytic degradation efficiency of CBZ with various control groups (data adapted from panel C).

An organic dye, direct violet 51 (DV51), was selected as the substrate to test the proposed concept. A typical catalytic reaction was conducted as follows: 5 mL of solution containing 3 mg/L Au_18_-D@F127 nanoparticles and 10 μM DV51, as well as 2 mM (NH_4_)_2_S_2_O_8_ was added to a 20 mL round bottle flask. The solution pH was controlled at 7 using an acetic acid/sodium acetate buffer solution. The reaction was carried out at mild stirring (300 rpm), with a 5 W 650 nm red LED providing irradiation. The DV51 removal efficiency was evaluated by measuring the OD at 550 nm (Figure 6A) of the prepared mixture. As shown in Figure 6B, Au_18_-D@F127 nanoparticles efficiently removed DV51 within 75 min (>80%), whereas all the control experiments showed less than 20% removal in the same period. The results from the control experiments confirmed that both light irradiation and gold NCs are crucial to achieve high efficiency dye removal. To quantify the catalytic performance, the DV51 degradation kinetic was fitted to a pseudo-first-order reaction model.^63^ As shown in Figure S10, the apparent rate constant (*k*) of DV51 degradation with the 650 nm + S_2_O_8_^2–^ + Au_18_-D@F127 conditions is 0.02 min^–1^ (*R*^2^ = 0.99), nearly 13 times higher than that of the control experiment (i.e., without Au_18_-D@F127, *k* = 0.0016 min^–1^).

Furthermore, we tested the photodegradation of another biorecalcitrant microcontaminant carbamazepine (CBZ) with the developed system.^64^ In the experiment, the concentration of CBZ was set to 25 μM, with all other parameters kept the same as in the previous experiments, and an 8 W 365 nm UV lamp was used to provide irradiation. Since other ingredients also exhibit nontrivial absorbance at the characteristic absorption peak wavelength (280 nm) of CBZ, the initial absorbance of CBZ (*A*_0_, before mixing with any other substances) was recorded, and the degradation efficiency was calculated by . As shown in Figure 6C, 85% of the initial CBZ can be efficiently degraded within 15 min while other contrast groups show much less activity (Figure 6D). UV–vis analysis (Figure S11A) shows that Au_18_-D@F127 NCs within the polymer micelle remain intact during the photocatalytic degradation process, indicating its high stability under UV light and sulfate radicals. A cyclic photocatalytic test (Figure S11B) confirmed the stability of the photocatalyst over three reaction cycles. The rate constant *k* of CBZ degradation with the 365 nm + Au_18_-D@F127 + S_2_O_8_^2–^ is calculated to be 0.12 min^–1^ (Figure S12), about 6 times higher than that of the control group (without Au_18_-D@F127). Of note, the control experiment with the less stable Au_18_(CHT)_14_@F127 showed that the NCs decomposed under similar catalytic conditions.

It is worth noting that F127 is stable in the strong oxidation of sulfate radicals, because otherwise, Au_18_-D would precipitate out of water due to Au_18_-D hydrophobicity without F127 coating. Throughout the catalytic reaction, no precipitation was seen, and the optical absorption spectrum of Au_18_-D and its peak intensity remained stable (Figure S11A); thus, F127 and Au_18_-D are both stable in the photocatalytic process.

### DFT Simulations of the Absorption Spectrum and Mechanism of S_2_O_8_^2–^ Decomposition

**Figure 7:**
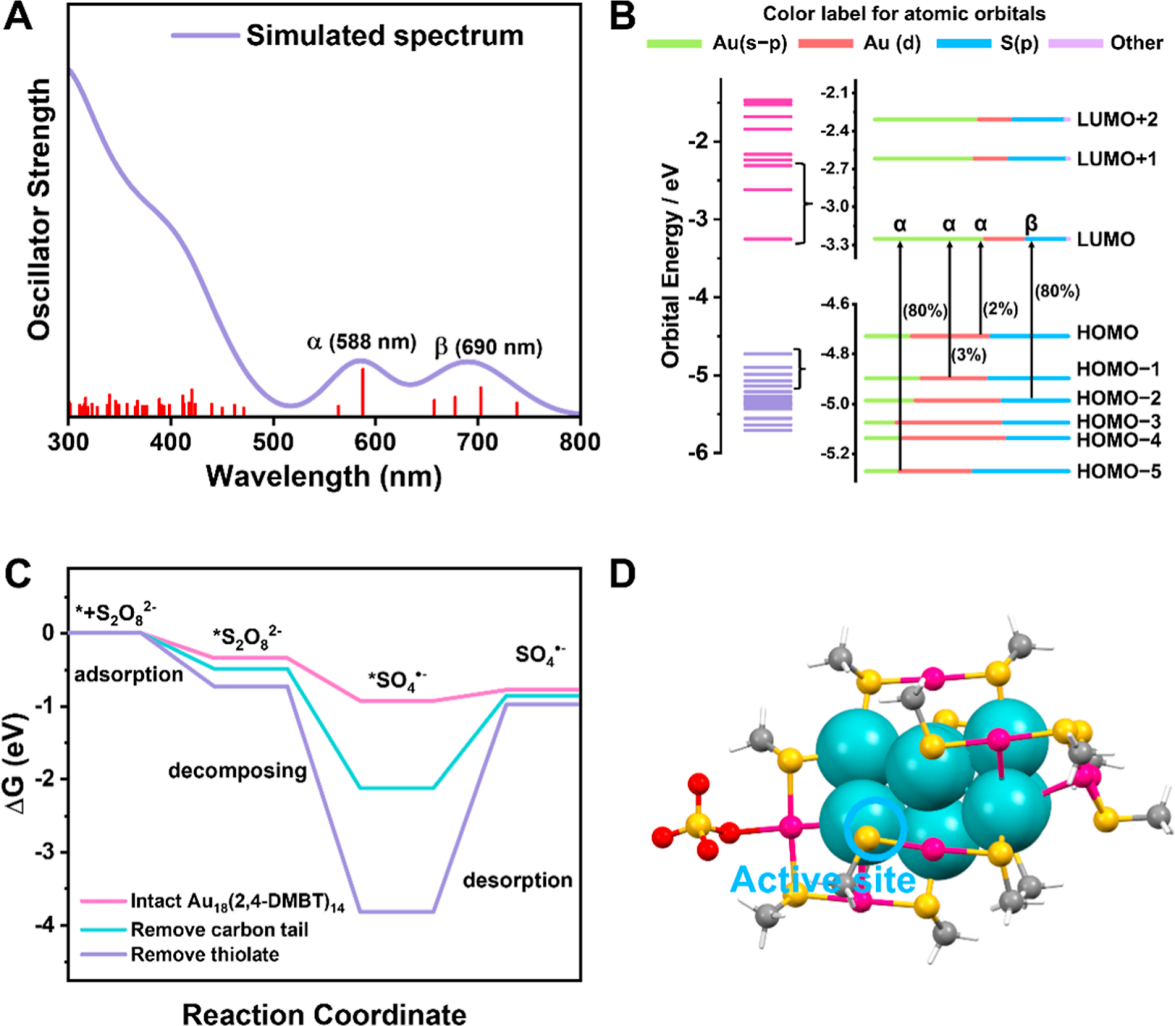
(A) Time dependent DFT-simulated absorption spectrum of Au_18_(SCH_3_)_14_. (B) Kohn–Sham (KS) orbital energy level diagram of Au_18_(SCH_3_)_14_. (C) Calculated free energy profile for the S_2_O_8_^2–^ to SO_4_^•–^ process on Au_18_(SCH_3_)_14_. The asterisk (*) represents the adsorption sites. (D) DFT-simulated catalytic active site on Au_18_(SCH_3_)_14_ for the activation of *S_2_O_8_^2–^ to form *SO_4_^•–^.

The absorption spectrum was calculated for the optimized ground-state structure of Au_18_(SR)_14_ at the time-dependent DFT level. The initial atomic coordinates were taken from single-crystal X-ray diffraction data with the –R group replaced by –CH_3_ for computational efficiency. The simulated absorption spectrum (Figure 7A) shows good agreement with the experimental results, all with two discernible characteristic absorption peaks above 500 nm. The Kohn–Sham molecular orbitals, energies, and atomic orbital contributions are depicted in Figure 7B. In the electronic structure of Au_18_(SCH_3_)_14_, the LUMO is mainly composed of 6sp atomic orbitals of gold, while the HOMO through HOMO – 5 orbitals are primarily constructed from the 5d^10^ atomic orbitals. The two characteristic absorption peaks of Au_18_ are attributed to transitions from HOMO – 5 to LUMO (α transition, 588 nm) and HOMO – 2 to LUMO (β transition, 690 nm), respectively.

DFT calculations were used to elucidate the decomposition pathways of the S_2_O_8_^2–^/pollutant catalyzed by Au_18_(SR)_14_. The role of photoexcitation is accounted for by applying the delta self-consistent field approach (ΔSCF) (see Supporting Information for the description of methodology).^65^ This approximation was previously used to simulate reactions on the excited state potential energy surface.^66−68^ Three potential reaction centers were explored: (i) the surface Au atom of the intact Au_18_ NC, (ii) the exposed S atom after the removal of a –CH_3_ group, and (iii) the exposed Au atom after the removal of an –SCH_3_ group (Figure S13). The degradation process was assumed to take place through the following elementary steps:^63^ adsorption of *S_2_O_8_^2–^ (where, * indicates adsorption), radical adduct decomposition, and *SO_4_^•–^ desorption. The corresponding predicted reaction Gibbs free energy (Δ*G*) profiles are shown in Figure 7C. For all of the considered reaction centers, the adsorption of *S_2_O_8_^2–^ and the subsequent cleavage of the O–O bond of *S_2_O_8_^2–^ to form *SO_4_^•–^ are both exothermic processes, indicating that both steps are thermodynamically downhill processes. The decomposition energy of *S_2_O_8_^2–^ is determined to be Δ*G* = −0.93, −2.14, and −3.83 eV on the intact NC, demethylated S, and dethiolated Au. The release of *SO_4_^•–^ is predicted to be the rate-determining (thermodynamic limiting step), being endothermic among all of the elementary steps. The desorption energy of *SO_4_^•–^ at the surface of the intact NC, is found to be slightly uphill, while significantly higher release energy is required on partially ligand protected counterparts. Therefore, the intact Au_18_ NC without any ligand removal (Figure 7D) is predicted to be the active species for this photocatalytic process,^69,70^ in contrast to the typical ligand removal scenario in electrochemical catalytic reaction.^71−73^ Overall, by accelerating the rate-limiting step, the desorption ability of sulfate radicals at the surface of the intact Au_18_ NC is enhanced, facilitating the interaction of sulfate radicals with pollutants and accelerating the degradation process of pollutants.

## Conclusions

In summary, this work presents a generalizable strategy for transferring organic soluble gold NCs into the aqueous phase and an application for robust photocatalysis in water. Specifically, we first synthesized and solved the structure of Au_18_(DMBT)_14_ and used this product to demonstrate the phase transfer and photocatalytic application. The aromatic ligand endows Au_18_(DMBT)_14_ with excellent antioxidation capability and photostability (even under 365 nm UV). Using a Pluronic F127-assisted method, organic-soluble Au_18_(DMBT)_14_ is successfully transferred into the aqueous phase without altering its structure and electronic and optical features. Interestingly, the resulting Au_18_-D@F127 nanoparticles (<2.5 nm core diameter, wrapping up to several Au_18_ NCs per nanoparticle) showed a 10-fold increase in PL intensity, unlike quantum dots that lead to severe quenching upon transfer to aqueous solution. Additionally, the Au_18_-D@F127 nanoparticles can efficiently activate persulfate ions for the photocatalytic degradation of organic pollutants in water. DFT simulations indicate that the surface metal site in intact Au_18_(DMBT)_14_ serves as the reaction site for the catalytic process. The Pluronic F127-assisted method is generalized to other thiolate-protected gold NCs, converting them from weakly to moderately emissive in PL. Their tunable optical properties and stronger emission in the near-infrared range render these aqueous NCs quite promising for future developments in medical imaging technologies and biosensing platforms, as well as solar energy conversion.

## Methods/Experimental Section

The syntheses of Au_18_(CHT)_14_, Au_18_(DMBT)_14_, Au_38_(DMBT)_24_, Au_28_(*p*-MBT)_20_, Au_36_(*p*-MBT)_24_, Au_44_(*p*-MBT)_28_, Au_52_(*p*-MBT)_32_, and Au_42_(PET)_32_ NCs, as well as F127 polymer wrapping of Au NCs for organic-to-aqueous phase transfer, are provided in the Supporting Information. Spectroscopic characterization includes optical absorption, steady-state, and time-resolved PL. Imaging of the NCs was conducted by STEM. Photocatalytic degradation of organic waste dyes was performed in aqueous solutions. DFT simulations were also carried out. Details are provided in the Supporting Information.

## Supporting Information Available

Details of the synthesis of NCs, spectroscopic measurements, X-ray crystallography, STEM imaging, photocatalysis, and theoretical simulations (PDF)
Data for Au_18_(DMBT)_14_ (CIF)

## Supplementary Material

nn4c18197_si_001.pdf

nn4c18197_si_002.cif

## References

[ref1] Jin R.; Zeng C.; Zhou M.; Chen Y. Atomically Precise Colloidal Metal Nanoclusters and Nanoparticles: Fundamentals and Opportunities. Chem. Rev. 2016, 116, 10346–10413. 10.1021/acs.chemrev.5b00703. 27585252

[ref2] Kang X.; Zhu M. Tailoring the Photoluminescence of Atomically Precise Nanoclusters. Chem. Soc. Rev. 2019, 48, 2422–2457. 10.1039/C8CS00800K. 30838373

[ref3] Chakraborty I.; Pradeep T. Atomically Precise Clusters of Noble Metals: Emerging Link Between Atoms and Nanoparticles. Chem. Rev. 2017, 117, 8208–8271. 10.1021/acs.chemrev.6b00769. 28586213

[ref4] Li S.; Li N.-N.; Dong X.-Y.; Zang S.-Q.; Mak T. C. Chemical Flexibility of Atomically Precise Metal Clusters. Chem. Rev. 2024, 124, 7262–7378. 10.1021/acs.chemrev.3c00896. 38696258

[ref5] Takano S.; Tsukuda T. Chemically Modified Gold/Silver Superatoms as Artificial Elements at Nanoscale: Design Principles and Synthesis Challenges. J. Am. Chem. Soc. 2021, 143, 1683–1698. 10.1021/jacs.0c11465. 33481579

[ref6] Chakraborty P.; Nag A.; Chakraborty A.; Pradeep T. Approaching Materials with Atomic Precision Using Supramolecular Cluster Assemblies. Acc. Chem. Res. 2019, 52, 2–11. 10.1021/acs.accounts.8b00369. 30507167

[ref7] Wu Z.; Yao Q.; Chai O. J. H.; Ding N.; Xu W.; Zang S.; Xie J. Unraveling the Impact of Gold (I)–Thiolate Motifs on the Aggregation-Induced Emission of Gold Nanoclusters. Angew. Chem. 2020, 132, 10020–10025. 10.1002/ange.201916675. 32011796

[ref8] Zhang S.-S.; Havenridge S.; Zhang C.; Wang Z.; Feng L.; Gao Z.-Y.; Aikens C. M.; Tung C.-H.; Sun D. Sulfide Boosting Near-Unity Photoluminescence Quantum Yield of Silver Nanocluster. J. Am. Chem. Soc. 2022, 144, 18305–18314. 10.1021/jacs.2c06093. 36169057

[ref9] Biswas S.; Das A. K.; Mandal S. Surface Engineering of Atomically Precise M (I) Nanoclusters: From Structural Control to Room Temperature Photoluminescence Enhancement. Acc. Chem. Res. 2023, 3721–3727. 10.1021/acs.accounts.3c00176 37357739

[ref10] Shen H.; Deng G.; Kaappa S.; Tan T.; Han Y. Z.; Malola S.; Lin S. C.; Teo B. K.; Häkkinen H.; Zheng N. Highly Robust but Surface-Active: An N-Heterocyclic Carbene-Stabilized Au_25_ Nanocluster. Angew. Chem. 2019, 131, 17895–17899. 10.1002/ange.201908983. 31517436

[ref11] Pyo K.; Thanthirige V. D.; Kwak K.; Pandurangan P.; Ramakrishna G.; Lee D. Ultrabright Luminescence from Gold Nanoclusters: Rigidifying the Au (I)–Thiolate Shell. J. Am. Chem. Soc. 2015, 137, 8244–8250. 10.1021/jacs.5b04210. 26061198

[ref12] Pniakowska A.; Kumaranchira Ramankutty K.; Obstarczyk P.; Perić Bakulić M.; Sanader Maršić Ž.; Bonačić-Koutecký V.; Bürgi T.; Olesiak-Bańska J. Gold-Doping Effect on Two-Photon Absorption and Luminescence of Atomically Precise Silver Ligated Nanoclusters. Angew. Chem., Int. Ed. 2022, 61, e202209645 10.1002/anie.202209645. 36005739

[ref13] Bootharaju M. S.; Kozlov S. M.; Cao Z.; Shkurenko A.; El-Zohry A. M.; Mohammed O. F.; Eddaoudi M.; Bakr O. M.; Cavallo L.; Basset J.-M. Tailoring the Crystal Structure of Nanoclusters Unveiled High Photoluminescence via Ion Pairing. Chem. Mater. 2018, 30, 2719–2725. 10.1021/acs.chemmater.8b00328.

[ref14] Zhao T.; Herbert P. J.; Zheng H.; Knappenberger K. L. State-Resolved Metal Nanoparticle Dynamics Viewed through the Combined Lenses of Ultrafast and Magneto-optical Spectroscopies. Acc. Chem. Res. 2018, 51, 1433–1442. 10.1021/acs.accounts.8b00096. 29738235

[ref15] Higaki T.; Russell J. C.; Paley D. W.; Roy X.; Jin R. Electron Transport Through Supercrystals of Atomically Precise Gold Nanoclusters: a Thermal Bi-Stability Effect. Chem. Sci. 2023, 14, 13191–13197. 10.1039/D3SC02753H. 38023517 PMC10664525

[ref16] Liu Z.; Luo L.; Jin R. Visible to NIR-II Photoluminescence of Atomically Precise Gold Nanoclusters. Adv. Mater. 2024, 36, 2309073 10.1002/adma.202309073. 37922431

[ref17] Luo L.; Liu Z.; Kong J.; Gianopoulos C. G.; Coburn I.; Kirschbaum K.; Zhou M.; Jin R. Three-Atom-Wide Gold Quantum Rods with Periodic Elongation and Strongly Polarized Excitons. Proc. Natl. Acad. Sci. U.S.A. 2024, 121, e2318537121 10.1073/pnas.2318537121. 38412123 PMC10927531

[ref18] Luo Z.; Yuan X.; Yu Y.; Zhang Q.; Leong D. T.; Lee J. Y.; Xie J. From Aggregation-Induced Emission of Au (I)–Thiolate Complexes to Ultrabright Au(0)@Au (I)–Thiolate Core–Shell Nanoclusters. J. Am. Chem. Soc. 2012, 134, 16662–16670. 10.1021/ja306199p. 22998450

[ref19] Yang G.; Mu X.; Pan X.; Tang Y.; Yao Q.; Wang Y.; Jiang F.; Du F.; Xie J.; Zhou X.; et al. Ligand Engineering of Au_44_ Nanoclusters for NIR-II Luminescent and Photoacoustic Imaging-Guided Cancer Photothermal Therapy. Chem. Sci. 2023, 14, 4308–4318. 10.1039/d2sc05729h. 37123188 PMC10132122

[ref20] Yao Q.; Liu L.; Malola S.; Ge M.; Xu H.; Wu Z.; Chen T.; Cao Y.; Matus M. F.; Pihlajamäki A.; et al. Supercrystal Engineering of Atomically Precise Gold Nanoparticles Promoted by Surface Dynamics. Nat. Chem. 2023, 15, 230–239. 10.1038/s41557-022-01079-9. 36357788

[ref21] Yang G.; Wang Z.; Du F.; Jiang F.; Yuan X.; Ying J. Y. Ultrasmall Coinage Metal Nanoclusters as Promising Theranostic Probes for Biomedical Applications. J. Am. Chem. Soc. 2023, 145, 11879–11898. 10.1021/jacs.3c02880. 37200506

[ref22] Yang G.; Pan X.; Feng W.; Yao Q.; Jiang F.; Du F.; Zhou X.; Xie J.; Yuan X. Engineering Au_44_ Nanoclusters for NIR-II Luminescence Imaging-Guided Photoactivatable Cancer Immunotherapy. ACS Nano 2023, 17, 15605–15614. 10.1021/acsnano.3c02370. 37503901

[ref23] Goswami N.; Yao Q.; Luo Z.; Li J.; Chen T.; Xie J. Luminescent Metal Nanoclusters with Aggregation-Induced Emission. J. Phys. Chem. Lett. 2016, 7, 962–975. 10.1021/acs.jpclett.5b02765. 26912457

[ref24] Song X.-R.; Goswami N.; Yang H.-H.; Xie J. Functionalization of Metal Nanoclusters for Biomedical Applications. Analyst 2016, 141, 3126–3140. 10.1039/C6AN00773B. 27146244

[ref25] Li Q.; Zhou M.; So W. Y.; Huang J.; Li M.; Kauffman D. R.; Cotlet M.; Higaki T.; Peteanu L. A.; Shao Z.; Jin R. A Mono-Cuboctahedral Series of Gold Nanoclusters: Photoluminescence Origin, Large Enhancement, Wide Tunability, and Structure–Property Correlation. J. Am. Chem. Soc. 2019, 141, 5314–5325. 10.1021/jacs.8b13558. 30860834

[ref26] Li Q.; Zeman C. J. IV; Ma Z.; Schatz G. C.; Gu X. W. Bright NIR-II Photoluminescence in Rod-Shaped Icosahedral Gold Nanoclusters. Small 2021, 17, 2007992 10.1002/smll.202007992. 33620777

[ref27] Li Q.; Zeman IV C. J.; Schatz G. C.; Gu X. W. Source of Bright Near-Infrared Luminescence in Gold Nanoclusters. ACS Nano 2021, 15, 16095–16105. 10.1021/acsnano.1c04759. 34613697

[ref28] Luo L.; Liu Z.; Du X.; Jin R. Near-Infrared Dual Emission from the Au_42_(SR)_32_ Nanocluster and Tailoring of Intersystem Crossing. J. Am. Chem. Soc. 2022, 144, 19243–19247. 10.1021/jacs.2c09107. 36239690

[ref29] Liu Z.; Luo L.; Kong J.; Kahng E.; Zhou M.; Jin R. Bright Near-Infrared Emission from the Au_39_(SR)_29_ Nanocluster. Nanoscale 2024, 16, 7419–7426. 10.1039/D4NR00677A. 38529816

[ref30] Shi W.-Q.; Zeng L.; He R.-L.; Han X.-S.; Guan Z.-J.; Zhou M.; Wang Q.-M. Near-Unity NIR Phosphorescent Quantum Yield from a Room-Temperature Solvated Metal Nanocluster. Science 2024, 383, 326–330. 10.1126/science.adk6628. 38236955

[ref31] Liu Z.; Zhou M.; Luo L.; Wang Y.; Kahng E.; Jin R. Elucidating the Near-Infrared Photoluminescence Mechanism of Homometal and Doped M_25_(SR)_18_ Nanoclusters. J. Am. Chem. Soc. 2023, 145, 19969–19981. 10.1021/jacs.3c06543. 37642696 PMC10510323

[ref32] Wang Y.; Liu Z.; Mazumder A.; Gianopoulos C. G.; Kirschbaum K.; Peteanu L. A.; Jin R. Tailoring Carbon Tails of Ligands on Au_52_(SR)_32_ Nanoclusters Enhances the Near-Infrared Photoluminescence Quantum Yield from 3.8 to 18.3%. J. Am. Chem. Soc. 2023, 145, 26328–26338. 10.1021/jacs.3c09846. 37982713 PMC10704554

[ref33] Kauffman D. R.; Alfonso D.; Matranga C.; Li G.; Jin R. Photomediated Oxidation of Atomically Precise Au_25_(SC_2_H_4_Ph)_18_^–^ Nanoclusters. J. Phys. Chem. Lett. 2013, 4, 195–202. 10.1021/jz302056q. 26291231

[ref34] Cui X.; Wang J.; Liu B.; Ling S.; Long R.; Xiong Y. Turning Au Nanoclusters Catalytically Active for Visible-Light-Driven CO_2_ Reduction through Bridging Ligands. J. Am. Chem. Soc. 2018, 140, 16514–16520. 10.1021/jacs.8b06723. 30407807

[ref35] Kurashige W.; Hayashi R.; Wakamatsu K.; Kataoka Y.; Hossain S.; Iwase A.; Kudo A.; Yamazoe S.; Negishi Y. Atomic-Level Understanding of the Effect of Heteroatom Doping of the Cocatalyst on Water-Splitting Activity in AuPd or AuPt Alloy Cluster-Loaded BaLa_4_Ti_4_O_15_. ACS Appl. Energy Mater. 2019, 2, 4175–4187. 10.1021/acsaem.9b00426.

[ref36] Isozaki K.; Iseri K.; Saito R.; Ueda K.; Nakamura M. Dual Catalysis of Gold Nanoclusters: Photocatalytic Cross-Dehydrogenative Coupling by Cooperation of Superatomic Core and Molecularly Modified Staples. Angew. Chem. 2024, 136, e202312135 10.1002/ange.202312135. 37926682

[ref37] Yan X.; Fu X. Y.; Xiao F. X. Filling the Gap: Atomically Precise Metal Nanoclusters-Induced Z-Scheme Photosystem toward Robust and Stable Solar Hydrogen Generation. Adv. Funct. Mater. 2023, 33, 2303737 10.1002/adfm.202303737.

[ref38] Hu J.; Yuan H.; Li P.; Wang J.; Liu Q.; Wang H.; Wang Q.; Yuan X. Synthesis and Photocatalytic Activity of ZnO-Au_25_ Nanocomposites. Sci. China:Chem. 2016, 59, 277–281. 10.1007/s11426-015-5487-6.

[ref39] Cao L.; Yang Y.; Zheng Y.; Cheng W.; Chen M.; Wang T.; Mu C.; Wu M.; Liu B. X-Ray-Triggered CO-Release from Gold Nanocluster: All-in-One Nanoplatforms for Cancer Targeted Gas and Radio Synergistic Therapy. Adv. Mater. 2024, 36, 2401017 10.1002/adma.202401017. 38573785

[ref40] Akiyama T.; Nagakawa H.; Tatsuma T. Well-Dispersed Au Co-Catalyst Deposited on a Rutile TiO_2_ Photocatalyst via Electron Traps. Phys. Chem. Chem. Phys. 2023, 25, 9031–9035. 10.1039/D2CP06064G. 36928706

[ref41] Weng B.; Lu K.-Q.; Tang Z.; Chen H. M.; Xu Y.-J. Stabilizing Ultrasmall Au Clusters for Enhanced Photoredox Catalysis. Nat. Commun. 2018, 9, 1543 10.1038/s41467-018-04020-2. 29670102 PMC5906565

[ref42] Tang J.; Xu N.; Ren A.; Ma L.; Xu W.; Han Z.; Chen Z.; Li Q. Two-Orders-of-Magnitude Enhancement of Photoinitiation Activity via a Simple Surface Engineering of Metal Nanoclusters. Angew. Chem., Int. Ed. 2024, 136, e202403645 10.1002/anie.202403645. 38530138

[ref43] Chen S.; Wang S.; Zhong J.; Song Y.; Zhang J.; Sheng H.; Pei Y.; Zhu M. The Structure and Optical Properties of the [Au_18_(SR)_14_] Nanocluster. Angew. Chem. 2015, 127, 3188–3192. 10.1002/ange.201410295. 25620108

[ref44] Das A.; Liu C.; Byun H. Y.; Nobusada K.; Zhao S.; Rosi N.; Jin R. Structure Determination of [Au_18_(SR)_14_]. Angew. Chem., Int. Ed. 2015, 54, 3140–3144. 10.1002/anie.201410161. 25619892

[ref45] Wang S.; Tang L.; Cai B.; Yin Z.; Li Y.; Xiong L.; Kang X.; Xuan J.; Pei Y.; Zhu M. Ligand Modification of Au_25_ Nanoclusters for Near-Infrared Photocatalytic Oxidative Functionalization. J. Am. Chem. Soc. 2022, 144, 3787–3792. 10.1021/jacs.2c01570. 35225599

[ref46] Liu Z.; Li Y.; Kahng E.; Xue S.; Du X.; Li S.; Jin R. Tailoring the Electron–Phonon Interaction in Au_25_(SR)_18_ Nanoclusters via Ligand Engineering and Insight into Luminescence. ACS Nano 2022, 16, 18448–18458. 10.1021/acsnano.2c06586. 36252530

[ref47] Li Y.; Juarez-Mosqueda R.; Song Y.; Zhang Y.; Chai J.; Mpourmpakis G.; Jin R. Ligand Exchange on Au_38_(SR)_24_: Substituent Site Effects of Aromatic Thiols. Nanoscale 2020, 12, 9423–9429. 10.1039/D0NR01430C. 32323691

[ref48] Zhou S.; Peng B.; Duan Y.; Liu K.; Ikkala O.; Ras R. H. Bright and Photostable Fluorescent Metal Nanocluster Supraparticles from Invert Emulsions. Angew. Chem., Int. Ed. 2022, 61, e202210808 10.1002/anie.202210808. PMC9804586 36045283

[ref49] Chen J.; Gu P.; Ran G.; Zhang Y.; Li M.; Chen B.; Lu H.; Han Y.-Z.; Zhang W.; Tang Z.; et al. Atomically Precise Photothermal Nanomachines. Nat. Mater. 2024, 23, 271–280. 10.1038/s41563-023-01721-y. 37957270

[ref50] Liu Q.; Yang T.; Feng W.; Li F. Blue-Emissive Upconversion Nanoparticles for Low-Power-Excited Bioimaging in vivo. J. Am. Chem. Soc. 2012, 134, 5390–5397. 10.1021/ja3003638. 22369318

[ref51] Zhou M.; Zeng C.; Sfeir M. Y.; Cotlet M.; Iida K.; Nobusada K.; Jin R. Evolution of Excited-State Dynamics in Periodic Au_28_, Au_36_, Au_44_, and Au_52_ Nanoclusters. J. Phys. Chem. Lett. 2017, 8, 4023–4030. 10.1021/acs.jpclett.7b01597. 28796513

[ref52] Zheng H.; Zhou Y.; Yan B.; Zhou G.; Cheng X.; Lin S.; Duan M.; Li J.; Wang L.; Fan C.; et al. DNA Framework-Guided Self-Limiting Aggregation for Highly Luminescent Metal Cluster Nanoaggregates. J. Am. Chem. Soc. 2024, 146, 17094–17102. 10.1021/jacs.4c02401. 38867462

[ref53] Baghdasaryan A.; Wang F.; Ren F.; Ma Z.; Li J.; Zhou X.; Grigoryan L.; Xu C.; Dai H. Phosphorylcholine-Conjugated Gold-Molecular Clusters Improve Signal for Lymph Node NIR-II Fluorescence Imaging in Preclinical Cancer Models. Nat. Commun. 2022, 13, 5613 10.1038/s41467-022-33341-6. 36153336 PMC9509333

[ref54] Bertorelle F.; Wegner K. D.; Perić Bakulić M.; Fakhouri H.; Comby-Zerbino C.; Sagar A.; Bernadó P.; Resch-Genger U.; Bonačić-Koutecký V.; Le Guével X.; Antoine R. Tailoring the NIR-II Photoluminescence of Single Thiolated Au_25_ Nanoclusters by Selective Binding to Proteins. Chem. - Eur. J. 2022, 28, e202200570 10.1002/chem.202200570. 35703399

[ref55] Zhang X. D.; Chen J.; Luo Z.; Wu D.; Shen X.; Song S. S.; Sun Y. M.; Liu P. X.; Zhao J.; Huo S.; Fan S.; Fan F.; Liang X.-J.; Xie J. Enhanced Tumor Accumulation of Sub-2 nm Gold Nanoclusters for Cancer Radiation Therapy. Adv. Healthcare Mater. 2014, 3, 133–141. 10.1002/adhm.201300189. 23873780

[ref56] Kawawaki T.; Mori Y.; Wakamatsu K.; Ozaki S.; Kawachi M.; Hossain S.; Negishi Y. Controlled Colloidal Metal Nanoparticles and Nanoclusters: Recent Applications as Cocatalysts for Improving Photocatalytic Water-Splitting Activity. J. Mater. Chem. A 2020, 8, 16081–16113. 10.1039/D0TA04750C.

[ref57] Wu G.; Mo Q.-L.; Xiao Y.; Wang K.; Ge X.-Z.; Xu S.-R.; Li J.-L.; Shao Y.-Q.; Xiao F.-X. Alloy Metal Nanocluster: a Robust and Stable Photosensitizer for Steering Solar Water Oxidation. Inorg. Chem. 2023, 62, 520–529. 10.1021/acs.inorgchem.2c03747. 36563080

[ref58] Abbas M. A.; Kamat P. V.; Bang J. H. Thiolated Gold Nanoclusters for Light Energy Conversion. ACS Energy Lett. 2018, 3, 840–854. 10.1021/acsenergylett.8b00070.

[ref59] Liu L.-J.; Zhang M.-M.; Deng Z.; Yan L.-L.; Lin Y.; Phillips D. L.; Yam V. W.-W.; He J. NIR-II Emissive Anionic Copper Nanoclusters with Intrinsic Photoredox Activity in Single-Electron Transfer. Nat. Commun. 2024, 15, 4688 10.1038/s41467-024-49081-8. 38824144 PMC11144245

[ref60] Zeng C.; Chen Y.; Kirschbaum K.; Lambright K. J.; Jin R. Emergence of Hierarchical Structural Complexities in Nanoparticles and Their Assembly. Science 2016, 354, 1580–1584. 10.1126/science.aak9750. 28008066

[ref61] Li G.; Abroshan H.; Liu C.; Zhuo S.; Li Z.; Xie Y.; Kim H. J.; Rosi N. L.; Jin R. Tailoring the Electronic and Catalytic Properties of Au_25_ Nanoclusters via Ligand Engineering. ACS Nano 2016, 10, 7998–8005. 10.1021/acsnano.6b03964. 27442235

[ref62] Koiki B. A.; Muzenda C.; Jayeola K. D.; Zhou M.; Marken F.; Arotiba O. A. Sulfate Radical in (Photo) electrochemical Advanced Oxidation Processes for Water Treatment: A Versatile Approach. J. Phys. Chem. Lett. 2023, 14, 8880–8889. 10.1021/acs.jpclett.3c01361. 37766606 PMC10561262

[ref63] Xie M.; Dai F.; Li J.; Dang X.; Guo J.; Lv W.; Zhang Z.; Lu X. Tailoring the Electronic Metal–Support Interactions in Supported Atomically Dispersed Gold Catalysts for Efficient Fenton-like Reaction. Angew. Chem. 2021, 133, 14491–14496. 10.1002/ange.202103652. 33843128

[ref64] Monteagudo J.; Durán A.; González R.; Expósito A. In situ Chemical Oxidation of Carbamazepine Solutions Using Persulfate Simultaneously Activated by Heat Energy, UV Light, Fe^2+^ Ions, and H_2_O_2_. Appl. Catal., B 2015, 176, 120–129. 10.1016/j.apcatb.2015.03.055.

[ref65] Hellman A.; Razaznejad B.; Lundqvist B. I. Potential-Energy Surfaces for Excited States in Extended Systems. J. Chem. Phys. 2004, 120, 4593–4602. 10.1063/1.1645787. 15267318

[ref66] Tacey S. A.; Szilvási T.; Xu L.; Schauer J. J.; Mavrikakis M. The Role of Iron-Oxide Aerosols and Sunlight in the Atmospheric Reduction of Hg (II) Species: A DFT+ U Study. Appl. Catal., B 2018, 234, 347–356. 10.1016/j.apcatb.2018.04.049.

[ref67] Kovačič Z. ˇ.; Likozar B.; Huš M. Ab Initio Modelling of Photocatalytic CO_2_ Reduction Reactions over Cu/TiO_2_ Semiconductors including the Electronic Excitation Effects. Chem. Eng. J. 2024, 485, 149894 10.1016/j.cej.2024.149894.

[ref68] Maurer R. J.; Reuter K. Assessing Computationally Efficient Isomerization Dynamics: ΔSCF Density-Functional Theory Study of Azobenzene Molecular Switching. J. Chem. Phys. 2011, 135, 224303 10.1063/1.3664305. 22168690

[ref69] Wang Y.; Wan X.-K.; Ren L.; Su H.; Li G.; Malola S.; Lin S.; Tang Z.; Hakkinen H.; Teo B. K.; et al. Atomically Precise Alkynyl-Protected Metal Nanoclusters as a Model Catalyst: Observation of Promoting Effect of Surface Ligands on Catalysis by Metal Nanoparticles. J. Am. Chem. Soc. 2016, 138, 3278–3281. 10.1021/jacs.5b12730. 26922997

[ref70] Wu Z.; Hu G.; Jiang D.-e.; Mullins D. R.; Zhang Q.-F.; Allard Jr L. F.; Wang L.-S.; Overbury S. H. Diphosphine-Protected Au_22_ Nanoclusters on Oxide Supports are Active for Gas-Phase Catalysis without Ligand Removal. Nano Lett. 2016, 16, 6560–6567. 10.1021/acs.nanolett.6b03221. 27685318

[ref71] Yang D.; Wang J.; Wang Q.; Yuan Z.; Dai Y.; Zhou C.; Wan X.; Zhang Q.; Yang Y. Electrocatalytic CO_2_ Reduction over Atomically Precise Metal Nanoclusters Protected by Organic Ligands. ACS Nano 2022, 16, 15681–15704. 10.1021/acsnano.2c06059. 36121680

[ref72] Wang J.; Xu F.; Wang Z. Y.; Zang S. Q.; Mak T. C. Ligand-Shell Engineering of a Au_28_ Nanocluster Boosts Electrocatalytic CO_2_ Reduction. Angew. Chem., Int. Ed. 2022, 61, e202207492 10.1002/anie.202207492. 35672264

[ref73] Deng G.; Yun H.; Bootharaju M. S.; Sun F.; Lee K.; Liu X.; Yoo S.; Tang Q.; Hwang Y. J.; Hyeon T. Copper Doping Boosts Electrocatalytic CO_2_ Reduction of Atomically Precise Gold Nanoclusters. J. Am. Chem. Soc. 2023, 145, 27407–27414. 10.1021/jacs.3c08438. 38055351

