# Supplementary material for: Generalizable Organic-to-Aqueous Phase Transfer of a Au18 Nanocluster with Luminescence Enhancement and Robust Photocatalysis in Water
Source: ACS Nano. 2025 Feb 28;19(9):9121–31. doi: 10.1021/acsnano.4c18197 (PMC11912569; doi:10.1021/acsnano.4c18197)
Supplement: Supplementary file 1 — nn4c18197_si_001.pdf [file nn4c18197_si_001.pdf]

(Supporting Information)

Generalizable Organic-to-Aqueous Phase Transfer of a Au_{18} Nanocluster with Luminescence Enhancement and Robust Photocatalysis in Water

Zhongyu Liu[1], Yitong Wang[1], Weijie Ji[1], Xiaowei Ma[1], Christopher G. Gianopoulos[2], Sebastian Calderon[3], Timothy Ma[1], Lianshun Luo[1], Abhrojyoti Mazumder[1], Kristin Kirschbaum[2], Elizabeth C. Dickey[3], Linda A. Peteanu,[1] Dominic Alfonso,[4]* and Rongchao Jin[1]*

[1]Department of Chemistry, Carnegie Mellon University, Pittsburgh, Pennsylvania 15213, USA

[2]Department of Chemistry and Biochemistry, University of Toledo, Toledo, Ohio 43606, USA

[3]Department of Materials Science and Engineering, Carnegie Mellon University, Pittsburgh, Pennsylvania 15213, USA

[4]National Energy Technology Laboratory, United States Department of Energy, Pittsburgh, Pennsylvania 15236, USA

*Correspondence authors, Email: Dominic.Alfonso@netl.doe.gov, rongchao@andrew.cmu.edu

Chemicals

Tetrachloroauric (III) acid ($HAuCl_4 \cdot 3H_2O$, 99.99% metal basis, Aldrich), 2,4-dimethylbenzenethiol (DMBT, C_8H_9SH, 98 %, Aldrich), cyclohexanethiol (CHT, 97 %, Aldrich), 2-phenylethanethiol (PET, 98 %, Aldrich), 4-methylbenzenethiol (*p*-MBT, 98 %, Aldrich), sodium borohydride ($NaBH_4$, Aldrich), tetraoctylammonium bromide (TOAB, ≥98 %, Fluka), carbamazepine (≥99%, Aldrich), direct violet 51 (Aldrich), Tetrabutylammonium hexafluorophosphate (98%, Aldrich), Phosphate buffered saline (PBS, Aldrich), triethylamine (Et_3N, 99%), tetrahydrofuran (HPLC grade, ≥99.9%, Aldrich), toluene (HPLC grade, ≥99.9%, Aldrich), dichloromethane (DCM, ACS reagent, ≥99.5%, Aldrich), acetonitrile (HPLC grade, ≥99.9%, Aldrich), methanol (MeOH, HPLC grade, ≥99.9 %, Aldrich), ethanol (EtOH, HPLC grade, ≥99.9 %, Aldrich), deuterium oxide (99.9% atom D, Aldrich). All chemicals were used as received without further purification. Deionized water was prepared with a Barnstead NANOpure Diamond system (18.2 MΩ cm). Thin-layer chromatography (TLC) plates were purchased from iChromatography (silica gel, 250 µm). A simulated biofluid (Biofluid & Solid Tissue Buffer) was purchased from Zymo Research Corporation.

Synthesis of $Au_{18}(DMBT)_{14}$

78.99 mg of $HAuCl_4 \cdot 3H_2O$ and 136.7 mg TOAB (0.25 mmol) were dissolved in 15 ml of THF under rapid stirring (~1000 rpm). The solution turned to deep orange in 30 minutes. Then, DMBT (138 µL) was added into the reaction mixture under ice bath. Subsequently, Et_3N (70 µL) was added all at once, and the stirring speed was reduced to ~100 rpm. After 30 min, a freshly prepared aqueous solution of $NaBH_4$ (47.5 mg, 1.25 mmol, 2 mL) was added dropwise to the reaction solution over a period of 5 minutes. Then, the stirring speed was raised to ~500 rpm. The reaction was allowed to proceed for 8 hours under 0 ^oC condition. After the reaction, the solvent was rotary evaporated, giving rise to a dark oil-like liquid. The oil was precipitated with methanol and the precipitate was washed by excess methanol. The obtained black product was further purified by thin layer chromatography (TLC, developing solvent: hexane:dichloromethane = 1:1(v/v)). The yield of $Au_{18}(DMBT)_{14}$ is ~5% based on gold atoms.

Crystallization of $Au_{18}(DMBT)_{14}$ was carried out by liquid phase diffusion of ethanol (3 mL) into a dichloromethane solution of $Au_{18}(DMBT)_{14}$ (1 mL, saturated) at room temperature. Dark green crystals were obtained after 4 days.

X-ray Crystallography Analysis of $Au_{18}(DMBT)_{14}$ Crystals

A small single crystal of good quality was found and used for X-ray crystallographic analysis, approximate dimensions 0.015 mm × 0.060 mm × 0.070 mm. The X-ray intensity data were measured using Cu-radiation, λ = 1.54178 Å.

A total of 2,380 frames were collected. The total exposure time was 34.89 hours. The frames were integrated with the Bruker SAINT software package using a narrow-frame algorithm. The integration of the data using a monoclinic unit cell yielded a total of 145,648 reflections to a maximum θ angle of 50.50° (1.00 Å resolution), of which 13,325 were independent (average redundancy 10.93, completeness = 98.2%, Rint = 16.67%, Rsig = 14.08%) and 7,664 (57.52%) were greater than $2\sigma(F2)$. The final cell constants of a = 24.0922(14) Å, b = 15.0663(8) Å, c = 35.6007(17) Å, β = 92.078(4) °, volume = 12,913.9(12) Å^3, are based upon the refinement of the XYZ-centroids of 9,948 reflections above 20 $\sigma(I)$ with 4.967° < 2θ < 100.0°. Data were scaled and corrected for absorption effects using the Multi-Scan method (SADABS). The ratio of minimum to maximum apparent transmission was 0.527.

The structure was solved and refined using the Bruker SHELXTL Software Package, in the space group $P2_1/c$, with Z = 4. All Au and most of the S-atoms were found using intrinsic phasing. All other atoms were located through successive Difference Fourier Syntheses. The 18 Au, and 14 S atoms were refined with anisotropic displacement parameters, while all C-atoms and the disordered Cl atoms were refined with isotropic displacement parameters. Two disordered lattice dichloromethane molecules could be identified. The occupancies of the solvent molecules were initially refined with a fixed Uiso, and then constrained to the refined value of 0.7, while the isotropic displacement parameters were allowed to be refined. Hydrogen atoms were placed in idealized positions and treated with a riding model. The final model consisted of 788 parameters with 0 restraints and converged to R1 = 9.25%, for the observed data with $I > 2\sigma(I)$, and wR2 = 16.85% for all data.

The largest peak in the final difference electron density synthesis was 1.357 e-/Å^3 and the largest hole was -1.635 e-/Å^3 with an RMS deviation of 0.283 e-/Å^3. On the basis of the final model, the calculated density was 2.871 g/cm^3 and F(000), 10,002 e^-.

Synthesis of $Au_{18}(CHT)_{14}$

The synthesis of crude $Au_{18}(CHT)_{14}$ NC followed a previously reported method.[1] Pure $Au_{18}(CHT)_{14}$ was separated by TLC (developing solvent: hexane:DCM = 2:1(v/v)). Pure $Au_{18}(CHT)_{14}$ was a grey band on the TLC plate (note: the crude product also contained $Au_{28}(CHT)_{20}$, an orange band on TLC).

Synthesis of Au_{52}(*p*-MBT)$_{32}$, Au_{44}(*p*-MBT)$_{28}$, Au_{36}(*p*-MBT)$_{24}$ and Au_{28}(*p*-MBT)$_{20}$

88.6 mg of $HAuCl_4\cdot 3H_2O$ (0.225 mmol) and 136.7 mg TOABr (0.25 mmol) were added into THF (15 mL). The mixture turned deep orange in 20 min. Then, *p*-MBT (146 mg, 1 mmol) was added to the mixture. The solution kept stirring for 60 minutes until the color of the mixture turned light yellow. Subsequently, $NaBH_4$ (30.4 mg, 0.8 mmol, dissolved in 5 mL of ice-cold nanopure water) was added to the mixture all at once. After 45 to 60 min of reaction, the dark organic phase was concentrated to 5 mL by rotary evaporation, and the aqueous phase was removed by glass pipette. After adding 2 mL DCM, excess methanol was added to the dark mixture, and the precipitates were separated by centrifugation. The dark solid was then washed

with methanol five times. The size-mixed crude NCs were extracted from the precipitates with a small amount of DCM and then dried. Further purification was conducted by PTLC (developing solvent: a mixture of DCM and *n*-hexane at 2:3 (v/v)). All four sizes can be separated at once.

Synthesis of $Au_{42}(PET)_{32}$

The synthesis of $Au_{42}(PET)_{32}$ followed the previously reported method.[2]

Synthesis of $Au_{38}(DMBT)_{24}$

$Au_{38}(DMBT)_{24}$ was a by-product (see **Figure S1**) from the synthesis of Au_{18}(2,4-DMBT)$_{14}$ and isolated by PTLC.

Organic-to-Aqueous Phase Transfer of Au nanoclusters

A 150 µM stock solution of Au nanoclusters in THF was first prepared. Next, 50 µL of this stock solution was mixed with 10 mg of Pluronic F127. Following this, 3 mL of water or heavy water were added to the mixture, which was then sonicated for 10 sec to ensure even dispersion. The mixture was subsequently placed in a 35 °C water bath and subjected to a nitrogen blow for 1 hour to evaporate THF. During the evaporation process, water or heavy water was added to maintain the mixture volume at a minimum of 1.5 mL. After the evaporation, additional water or heavy water was added to bring the total volume back to 3 mL. Note: an excess amount of F127 negatively affects the photocatalytic performance, thus, an optimal amount of F127 was used herein to balance water solubility of Au_{18}-D (short for $Au_{18}(DMBT)_{14}$)) and still maintain a good catalytic activity.

Photocatalytic Degradation of Organic Pollutants

For the photocatalytic degradation of direct violet 51 (DV51), a 5 mL aqueous solution containing 3 mg/L (on the Au_{18}-D basis) Au_{18}-D@F127 nanoparticles, 2.28 mg of $(NH_4)_2S_2O_8$ and 10 µM DV51 was prepared and added to a 20 mL round-bottom flask. The initial UV-vis spectrum was then measured. After a 30-minute equilibration period in the dark, a 5 W 650 nm red LED was turned on and the reaction was carried out with mild stirring at 300 rpm. The UV-vis absorption spectrum of the mixture was measured every 15 minutes.

For the photocatalytic degradation of carbamazepine (CBZ), the UV absorption spectrum of 25 µM CBZ aqueous solution was first recorded. Then, a 5 mL aqueous solution containing 3 mg/L (Au_{18}-D base) Au_{18}-D@F127 nanoparticles, 2.28 mg of $(NH_4)_2S_2O_8$ and 25 µM CBZ was prepared and added to a 20 mL round-bottom flask. The initial UV-vis spectrum of the mixture was then measured. After a 30-min equilibration period in the dark, an 8 W 365 nm UV lamp was turned on, and the UV-vis absorption spectrum of the mixture was measured every 5 minutes.

Steady-State UV-Vis-NIR Measurements

UV-Vis-NIR spectra of Au nanoclusters were collected with a UV-3600 Plus UV-VIS-NIR spectrophotometer (Shimadzu).

Steady-State and Time-Resolved Photoluminescence Measurements

Steady state photoluminescence spectra were measured on a FLS-1000 spectrofluorometer (Edinburgh). Near infrared PL was measured using a wide range InGaAs detector cooled by liquid nitrogen down to -

80 °C. Time-correlated single-photon counting (TCSPC) measurements were performed using an EPL-450 picosecond pulsed diode laser (Edinburgh Instruments). The excitation wavelength from EPL-450 was 450 nm with a variance smaller than 5 nm. The pulse duration was less than 100 ps.

PL Quantum Yield Measurements

A relative method was used ($Au_{25}(PET)_{18}^-$ as the reference, QY=1% in $CDCl_3$). The relative quantum yield (Φ_S) of the sample was calculated using:

$$\Phi_S = \Phi_R(\frac{I_S}{I_R})(\frac{1-10^{-A_R}}{1-10^{-A_S}})(\frac{n_S}{n_R})^2$$

where, Φ_R is the quantum yield of the reference standard, I is the integrated PL intensity, A is the absorbance of the solution at the excitation wavelength, and n is the refractive index of the solution solvent. (The subscripts S and R represent sample and reference, respectively.)

Scanning Transmission Electron Microscopy (STEM)

Characterization of drop-casted samples was conducted using a ThermoFisher Titan-Themis TEM operated at 200kV. High-angle annular dark-field (HAADF)-STEM images were acquired using a probe convergence angle of 17.9 mrads and a camera length that resulted in acceptance angles between 69 and 200 mrads. Energy-dispersive spectroscopy (EDS-STEM) analysis was performed using a Thermo Fisher SuperX detector, using a beam current of 150 pA, collected for 8 min.

Free Energy Profile Calculations

The Vienna Ab initio Simulation Package (VASP)[3-4] was deployed to carry out plane-wave density functional theory (DFT) calculations with the generalized gradient approximation (GGA) using the Perdew–Burke–Ernzerhof (PBE)[5] functional. The projected augmented wave (PAW)[6] was selected and a plane wave basis set having a kinetic energy cutoff of 520 eV. One-electron states were occupied following a Fermi–Dirac distribution with k_BT = 0.1 eV, and total energies were extrapolated to k_BT = 0 eV. The geometry optimizations were performed using a conjugate gradient algorithm with a force convergence smaller than 0.03 eV/Å. The $Au_{18}(SR)_{14}$ nanocluster model was derived from the experimentally solved crystal structures. The organic fragment of the ligands was modeled using a $-CH_3$ moiety to generate a computationally tractable model while accurately capturing the geometrical structure on the nanocluster. The resulting 88-atom $Au_{18}(SCH_3)_{14}$ model was inserted into a three-dimensional 30 Å × 30 Å × 30 Å periodic cubic box to exclude periodic interaction between them. The sampling of the Brillouin zone was conducted with a Γ-point *k*-point mesh.

Free energies of $S_2O_8^{2-}$ and SO_4^- radical adsorbates on the nanocluster and their counterpart gaseous phase species were calculated using statistical mechanics within the harmonic approximation. The reaction-free energies were calculated as follows: $\Delta G = \Delta E + \Delta ZPE + \int C_p\, dT - T\Delta S$ (T = 292 K), where ΔE is the electronic energy based on DFT calculations, while ΔZPE, C_p and ΔS are the zero-point energy corrections, heat capacity and entropy change, respectively. The vibrational frequencies were derived from hessians calculated from finite differences of analytic gradients on single molecules in vacuum or adsorbates. Only vibrational motion was considered for adsorbates on the nanocluster, while translational, rotational, and vibrational motions were all calculated for the gas-phase species.

In thermal reactions, the reaction happens on the ground state potential energy surface, which can be obtained with the aforementioned standard DFT free energy calculations. However, in the case of photocatalytic reactions, excited electrons must be accounted for. Here ΔSCF method was employed to

model reaction on excited state potential energy surface.[7-10] To estimate excitation energies, the ground state structures of the initial, intermediate and product states are first obtained. The molecular orbitals available for electrons from each ground state structure are then analyzed to identify the HOMO and LUMO. Using this information, an electron from HOMO is transferred to LUMO, and the excitation energy is calculated from the energy difference between this excited state and the ground state.

Calculations of Electronic Structure and UV-Vis Absorption Spectrum

Reliability and accuracy of the excitation energetics is enhanced via the time-dependent framework of DFT. The time-dependent density functional theory (TD-DFT), via the linear response scheme within the adiabatic approximation, was used to compute the UV-visible absorption spectrum of the optimized ground-state structure of model $Au_{18}(SCH_3)_{14}$. The Perdew-Burke-Ernzerhof (PBE) functional together with the def2-SV(P) basis set as implemented in TURBOMOLE 7.8 package[11] were chosen. Quadrature grids were of m4 quality.[12] The geometry optimization was started using the relaxed structure obtained from the plane-wave DFT calculations as implemented in VASP package. The model $Au_{18}(SCH_3)_{14}$ was relaxed without symmetry constraining with a quasi-Newton-Raphson structural optimizer. The TD-DFT calculations were performed with the ESCF module.[13] The computationally efficient resolution of identities approximation[14] was used to calculate 700 lowest singlet-to-singlet vertical excitations. Simulated spectra were generated by convolving the calculated absorption energies and intensities with a Gaussian function of sigma set to 40 nm coupled with sampling the energy over 700 points within a 300-800 nm range.

Electrochemistry

Differential pulse voltammetry of the aqueous Au_{18}-D@F127 nanoparticles was measured on a CHI 620C electrochemical station at room temperature. A platinum wire (the counter electrode), platinum working electrode, and Ag/AgCl quasi-reference electrode was used in the electrochemical test. $Au_{18}(DMBT)_{14}$ clusters (2.75 mg) with F-127 were dissolved in 5 mL phosphate buffered saline solution (PBS, pH = 7.4, containing 0.0027 mol/L potassium chloride, 0.137 mol/L sodium chloride, and 0.01 mol/L phosphate buffer). The solution was bubbled with dry N_2 for 30 min before the electrochemical measurements.

Differential pulse voltammetry of the organic soluble $Au_{18}(DMBT)_{14}$ was measured on a CHI 620C electrochemical station at room temperature. A platinum wire (the counter electrode), platinum working electrode, and Ag/AgCl quasi-reference electrode was used in the electrochemical test. $Au_{18}(DMBT)_{14}$ clusters (2.75 mg) and $TBAPF_6$ (0.1 mol/L) were dissolved in 5 mL DCM. The solution was bubbled with dry N_2 for 30 min before the electrochemical measurements.

Supporting Figures:

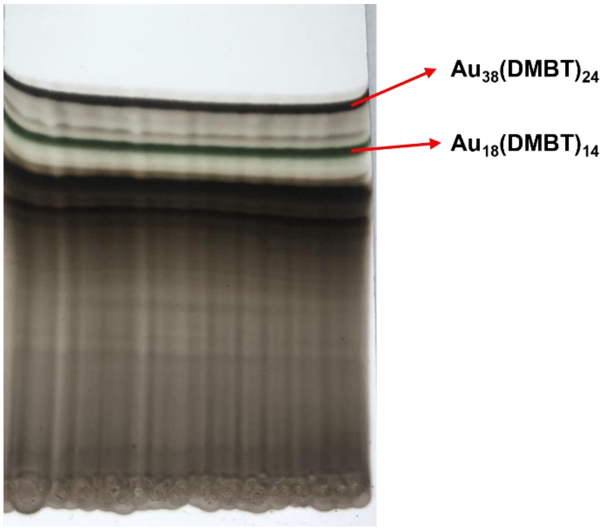

Figure S1. TLC separation of $Au_{18}(DMBT)_{14}$ from the product mixture.

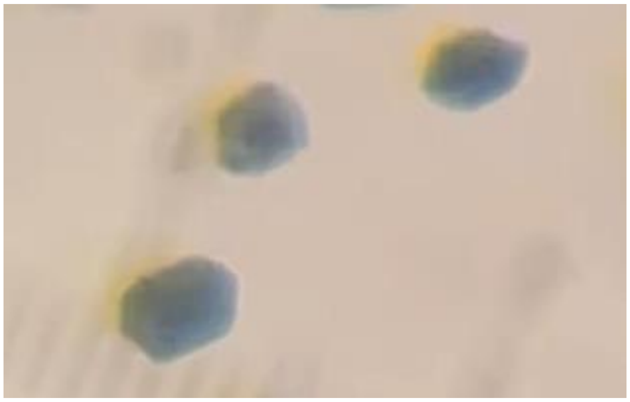

Figure S2. Photo micrograph of single crystals of $Au_{18}(DMBT)_{14}$.

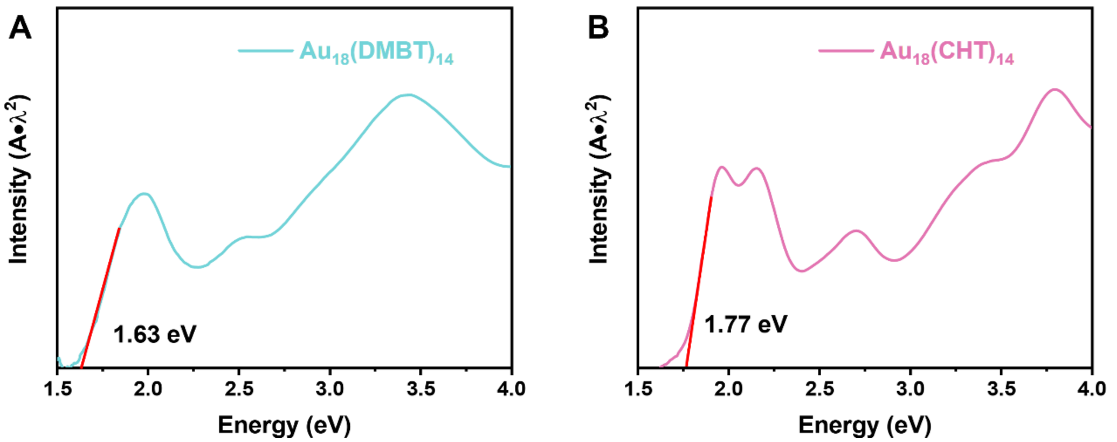

Figure S3. UV-vis spectrum of (A) $Au_{18}(DMBT)_{14}$ and (B) $Au_{18}(CHT)_{14}$ on photon energy scale. The energy gap was determined by extrapolating absorbance to zero (red straight lines).

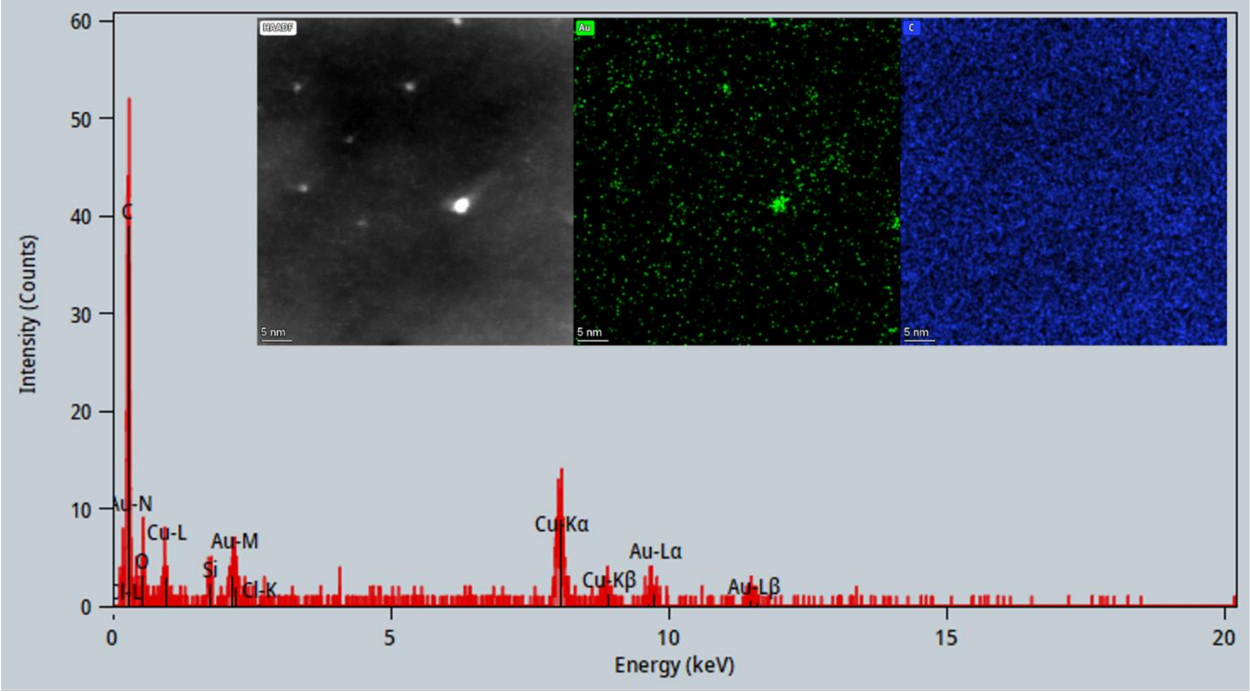

Figure S4. STEM-EDS characterization of Au_{18}-D@F127 nanoparticles, where the Cu X-ray signals (Cu-Kα, Cu-Kβ and Cu-L) come from the TEM grid. Inset images: HAADF-STEM image (left), and EDS mapping analysis of Au (middle) and C (right) on the same area of the STEM image. Note: Au_{18}-D = $Au_{18}(DMBT)_{14}$, and the Si and Cl EDS signals come from the grid, not from the sample.

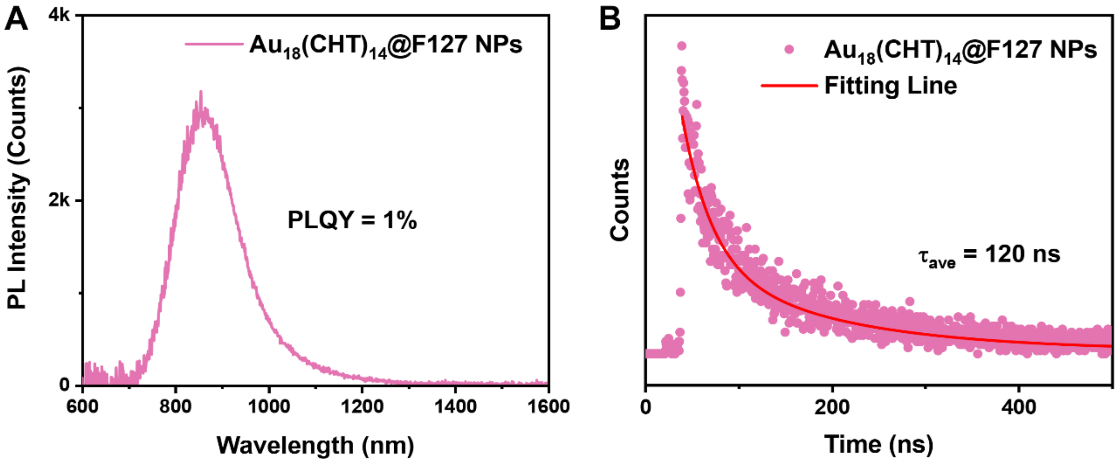

Figure S5. (A) PL spectra of $Au_{18}(CHT)_{14}$@F127 NPs in deaerated D_2O (with N_2). (B) PL decay curve of $Au_{18}(CHT)_{14}$@F127 NPs in D_2O.

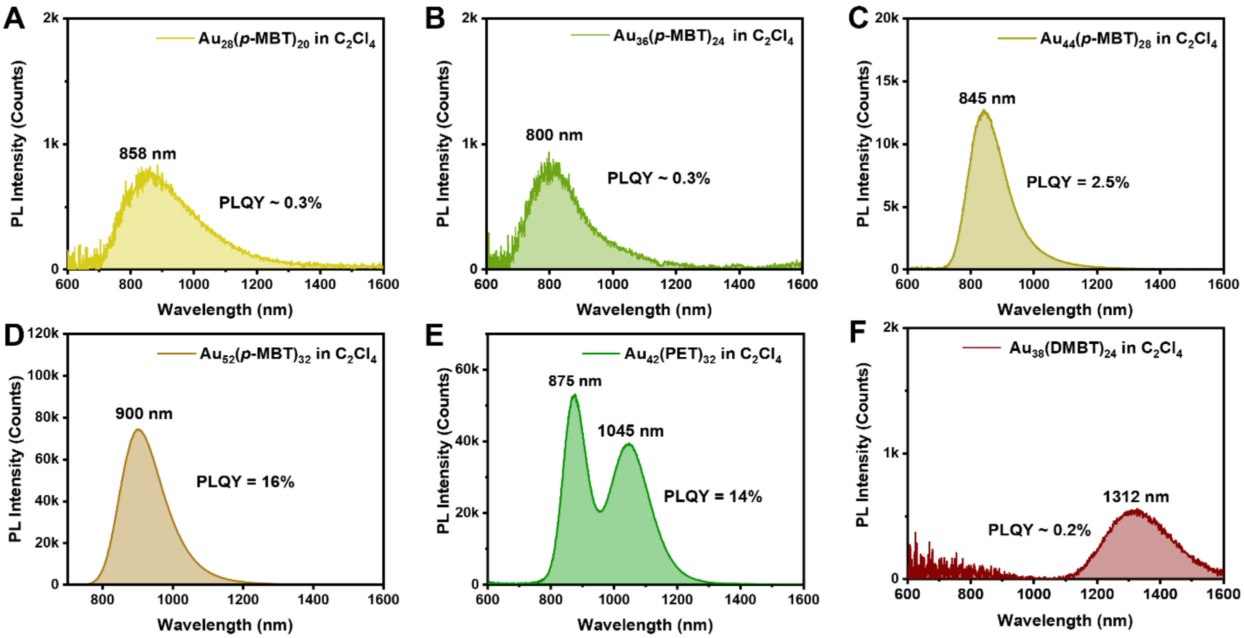

Figure S6. PL emission spectra of (A) Au_{28}(*p*-MBT)$_{20}$, (B) Au_{36}(*p*-MBT)$_{24}$, (C) Au_{44}(*p*-MBT)$_{28}$, (D) Au_{52}(*p*-MBT)$_{32}$, (E) $Au_{42}(PET)_{32}$ and (F) Au_{38}(2,4-DMBT)$_{24}$ in deaerated C_2Cl_4 (with N_2). (For PL measurements: excitation at 400 nm with 0.2 OD concentration, slit width 8 nm, and emission slit 8 nm.)

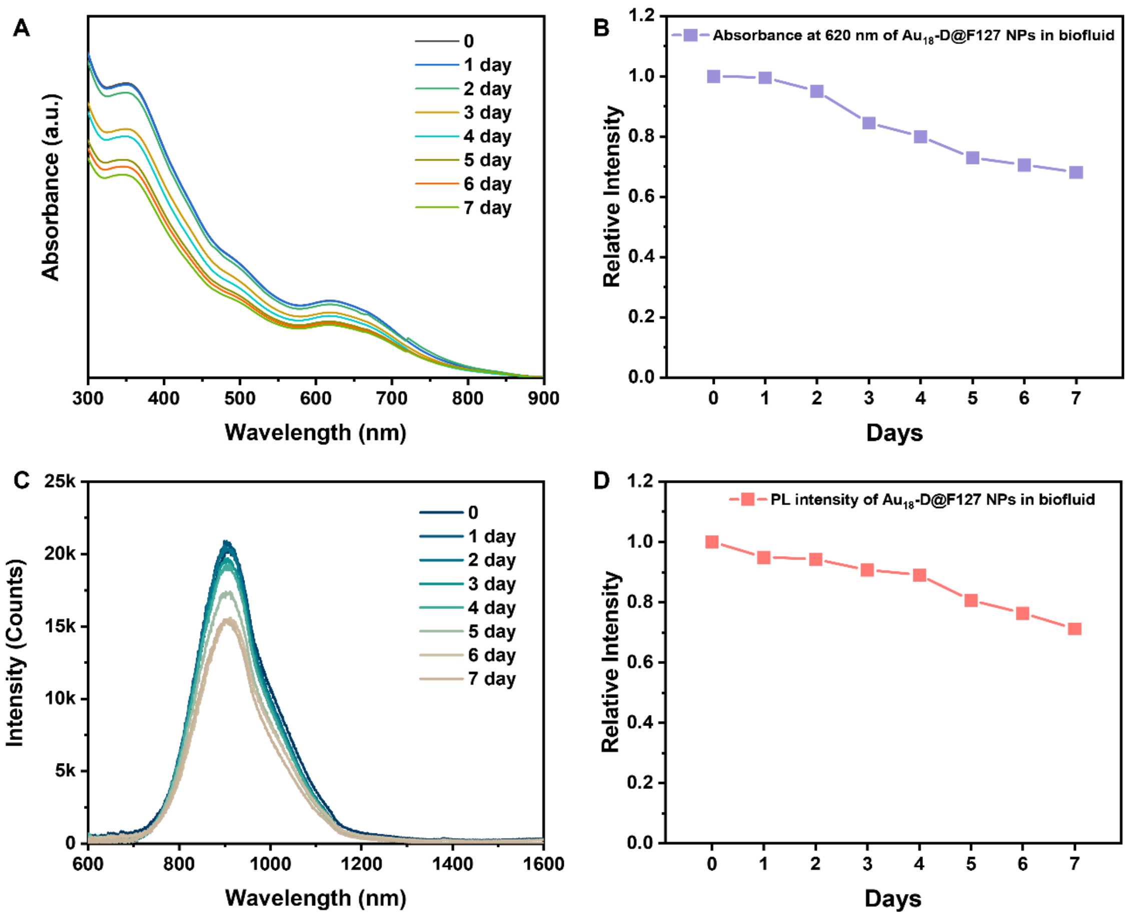

Figure S7. Photostability test of Au_{18}-D@F127 nanoparticles in a simulated biofluid. (A) UV-vis absorption spectral evolution, (B) change of absorbance at 620 nm. (C) PL spectral evolution, (D) change of PL intensity (the integrated peak area).

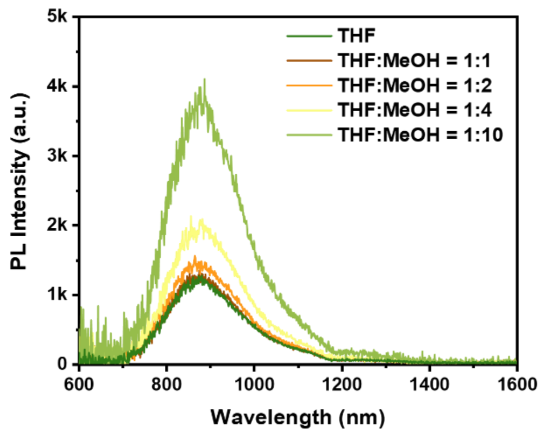

Figure S8. PL spectra of $Au_{18}(DMBT)_{14}$ in mixed THF/MeOH of different ratios (threefold enhancement of PL was obtained at MeOH/THF = 10/1).

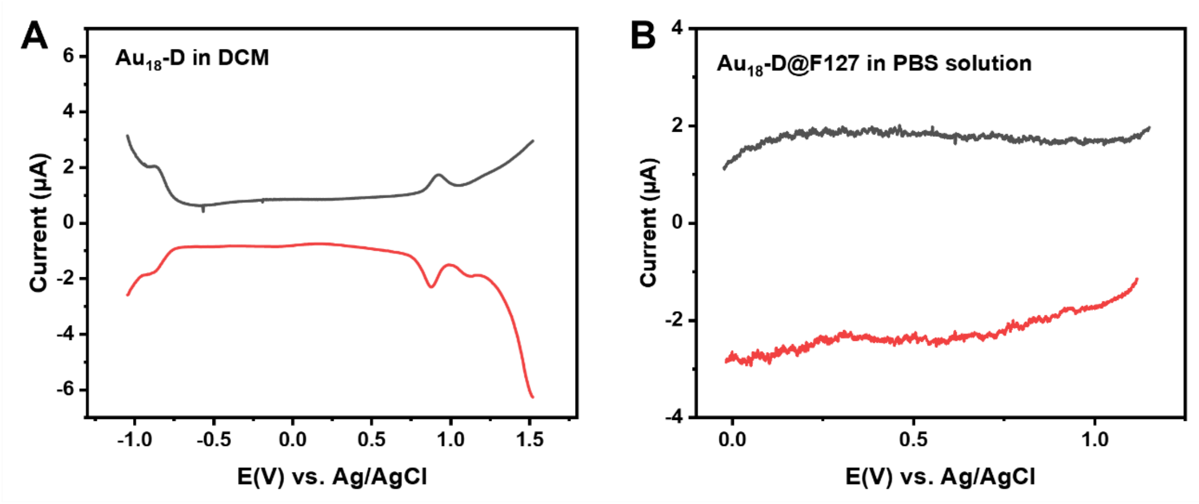

Figure S9. Differential pulse voltammetry tests of (A) Au_{18}-D clusters in DCM and (B) Au_{18}-D@F127 nanoparticles in phosphate buffered saline solution (PBS buffer).

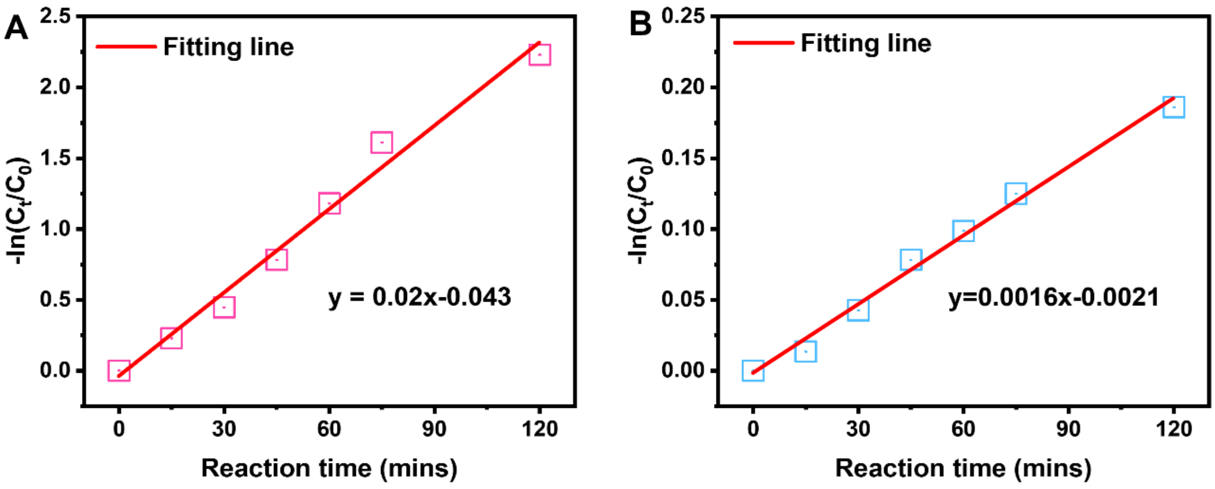

Figure S10. The degradation kinetics of direct violet 51 (A) 650 nm+$S_2O_8^{2-}$+Au_{18}-D@F127, (B) 650 nm+$S_2O_8^{2-}$.

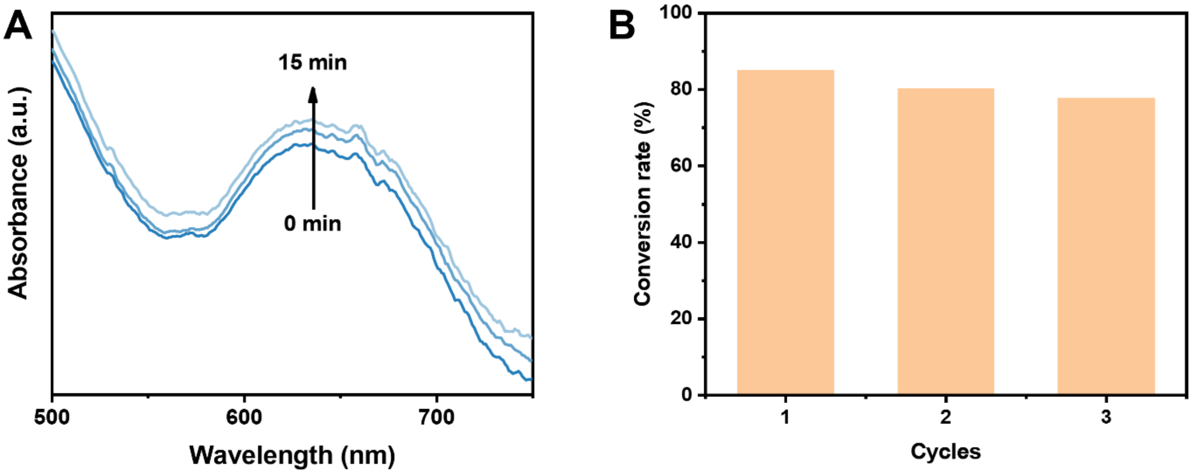

Figure S11. (A) Optical absorption features of $Au_{18}(DMBT)_{14}$ NCs remain the same during the photocatalytic reaction, indicating high stability. Note: the fluctuation of the baseline is due to small bubbles. (B) Conversion of carbamazepine in three cycles of the same Au_{18}-D@F127 catalyst.

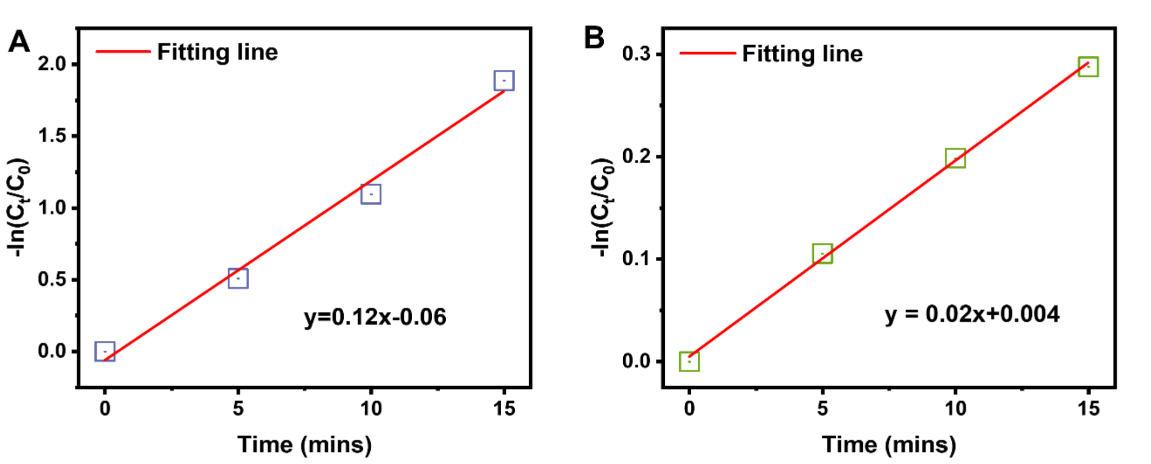

Figure S12. The degradation kinetics of carbamazepine (A) 365 nm+$S_2O_8^{2-}$+Au_{18}-D@F127, (B) 365 nm+$S_2O_8^{2-}$.

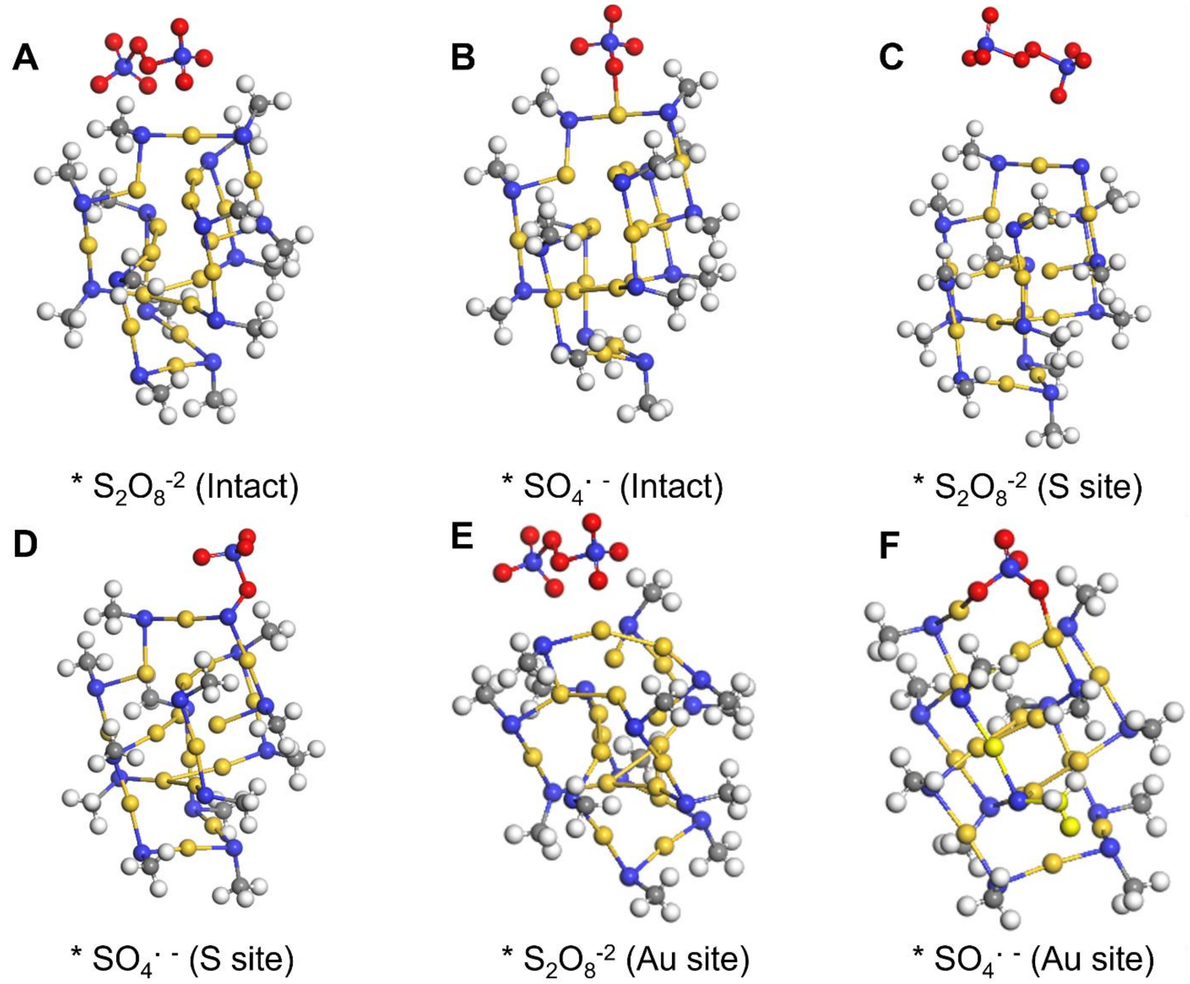

Figure S13. The adsorption and dissociation model of $S_2O_8^{2-}$ ion on intact Au_{18} NC (**A** and **B**), S site (**C** and **D**), and Au site (**E** and **F**).

Table S1. Sample and crystal data for $Au_{18}(2,4\text{-DMBT})_{14}$•$1.4(CH_2Cl_2)$.

**Chemical formula**	$C_{112}H_{125}Au_{18}S_{14}$ •$1.4(CH_2Cl_2)$	
**Formula weight**	5,582.83 g/mol	
**Temperature**	220(2) K	
**Wavelength**	1.54178 Å	
**Crystal size**	0.015 x 0.060 x 0.070 mm	
**Crystal system**	monoclinic	
**Space group**	$P2_1/c$	
**Unit cell dimensions**	a = 24.0922(14) Å	α = 90°
	b = 15.0663(8) Å	β = 92.078(4)°
	c = 35.6007(17) Å	γ = 90°
**Volume**	12,913.9(12) $Å^3$	
**Z**	4	
**Density (calculated)**	2.871 g/cm^3	
**Absorption coefficient**	40.314 mm^{-1}	
**F(000)**	10,002	

Table S2. Data collection and structure refinement for $Au_{18}(2,4\text{-DMBT})_{14}$•$1.4(CH_2Cl_2)$

**Theta range for data collection**	2.48 to 50.50°	
**Index ranges**	-23<=h<=23, -14<=k<=14, -35<=l<=32	
**Reflections collected**	14,5648	
**Independent reflections**	13,325 [R(int) = 0.1667]	
**Coverage of independent reflections**	98.2%	
**Absorption correction**	Multi-Scan	
**Max. and min. transmission**	0.2357 and 0.1243	
**Structure solution technique**	dual-space	
**Structure solution program**	XT, VERSION 2018/2	
**Refinement method**	Full-matrix least-squares on F^2	
**Refinement program**	SHELXL-2019/2 (Sheldrick, 2019)	
**Function minimized**	$\Sigma\ w(F_o^2 - F_c^2)^2$	
**Data / restraints / parameters**	13,325 / 0 / 788	
**Goodness-of-fit on F^2**	1.168	
**Δ/σ_{max}**	0.087	
**Final R indices**	7,664 data; I>2σ(I)	R1 = 0.0925, wR2 = 0.1479
	all data	R1 = 0.1678, wR2 = 0.1685
**Weighting scheme**	$w=1/[\sigma^2(F_o^2)+(0.0520P)^2]$<br>where $P=(F_o^2+2F_c^2)/3$	
**Largest diff. peak and hole**	1.357 and -1.635 $eÅ^{-3}$	
**R.M.S. deviation from mean**	0.283 $eÅ^{-3}$	

Table S3. Fitted lifetimes of PL of $Au_{18}(DMBT)_{14}$ and $Au_{18}(CHT)_{14}$. τ_{av} is the intensity-averaged lifetime: $\tau_{av} = \frac{\sum_i a_i \tau_i^2}{\sum_i a_i \tau_i}$

Sample	τ_1 (ns)	τ_2 (ns)	τ_{av} (ns)
$Au_{18}(DMBT)_{14}$	2 (a_1=29%)	13 (a_2=71%)	9
$Au_{18}(CHT)_{14}$	7 (20%)	25 (80%)	21

Table S4. Fitted lifetimes of PL of $Au_{18}(DMBT)_{14}$@F127 and $Au_{18}(CHT)_{14}$@F127. τ_{av} is the intensity-averaged lifetime.

Sample	τ_1 (ns)	τ_2 (ns)	τ_{av} (ns)
$Au_{18}(DMBT)_{14}$@F127	23 (21%)	183 (79%)	150
$Au_{18}(CHT)_{14}$@F127	31 (26%)	151 (74%)	120

Supporting References:

s1. Das, A.; Liu, C.; Byun, H. Y.; Nobusada, K.; Zhao, S.; Rosi, N.; Jin, R., Structure Determination of [$Au_{18}(SR)_{14}$]. *Angew. Chem. Int. Ed.* **2015,** *54*, 3140-3144.
s2. Luo, L.; Liu, Z.; Du, X.; Jin, R., Near-Infrared Dual Emission from the $Au_{42}(SR)_{32}$ Nanocluster and Tailoring of Intersystem Crossing. *J. Am. Chem. Soc.* **2022,** *144*, 19243-19247.
s3. Kresse, G.; Furthmüller, J., Efficiency of Ab-initio Total Energy Calculations for Metals and Semiconductors Using a Plane-wave Basis Set. *Comput. Mater. Sci.* **1996,** *6*, 15-50.
s4. Kresse, G.; Hafner, J., Ab initio Molecular-Dynamics Simulation of the Liquid-Metal–Amorphous-Semiconductor Transition in Germanium. *Phys. Rev. B* **1994,** *49*, 14251.
s5. Perdew, J. P.; Burke, K.; Ernzerhof, M., Generalized Gradient Approximation Made Simple. *Phys. Rev. Lett.* **1996,** *77*, 3865.
s6. Kresse, G.; Joubert, D., From Ultrasoft Pseudopotentials to the Projector Augmented-Wave Method. *Phys. Rev. B* **1999,** *59*, 1758.
s7. Hellman, A.; Razaznejad, B.; Lundqvist, B. I., Potential-Energy Surfaces for Excited States in Extended Systems. *J. Chem. Phys.* **2004,** *120*, 4593-4602.
s8. Tacey, S. A.; Szilvási, T.; Xu, L.; Schauer, J. J.; Mavrikakis, M., The Role of Iron-Oxide Aerosols and Sunlight in the Atmospheric Reduction of Hg (II) Species: A DFT+ U Study. *Appl. Catal. B* **2018,** *234*, 347-356.
s9. Kovačič, Ž.; Likozar, B.; Huš, M., Ab Initio Modelling of Photocatalytic CO_2 Reduction Reactions over Cu/TiO_2 Semiconductors including the Electronic Excitation Effects. *Chem. Eng. J.* **2024,** *485*, 149894.
s10. Maurer, R. J.; Reuter, K., Assessing Computationally Efficient Isomerization Dynamics: ΔSCF Density-Functional Theory Study of Azobenzene Molecular Switching. *J. Chem. Phys.* **2011,** *135*, 224303.
s11. Ahlrichs, R.; Bär, M.; Häser, M.; Horn, H.; Kölmel, C., Electronic Structure Calculations on Workstation Computers: The Program System Turbomole. *Chem. Phys. Lett.* **1989,** *162*, 165-169.
s12. Treutler, O.; Ahlrichs, R., Efficient Molecular Numerical Integration Schemes. *J. Chem. Phys.* **1995,** *102*, 346-354.
s13. Bauernschmitt, R.; Ahlrichs, R., Treatment of Electronic Excitations within the Adiabatic Approximation of Time Dependent Density Functional Theory. *Chem. Phys. Lett.* **1996,** *256*, 454-464.
s14. Weigend, F.; Häser, M., RI-MP2: First Derivatives and Global Consistency. *Theor. Chem. Acc.* **1997,** *97*, 331-340.
